# A novel rabbit model of early osteoarthritis exhibits gradual cartilage degeneration after medial collateral ligament transection outside the joint capsule

**DOI:** 10.1038/srep34423

**Published:** 2016-10-19

**Authors:** Zhenlong Liu, Xiaoqing Hu, Zhentao Man, Jiying Zhang, Yanfang Jiang, Yingfang Ao

**Affiliations:** 1 Institute of Sports Medicine, Beijing Key Laboratory of Sports Injuries, Peking University Third Hospital, 49 North Garden Road, Haidian District, Beijing 100191, People’s Republic of China

## Abstract

Though many surgical animal models have been used to induce osteoarthritis (OA) of the knee joint, they always open the capsule of the joint. Any surgical procedures that incises the capsule may cause inflammation, pain, and possibly altered gait. One common disadvantage of these surgically induced animal models is that they may affect the initial structures and synovial fluid in joint. These animal models may not be suitable for research into synovial fluid changes during early OA. This study aimed to create an animal model of early OA by resecting the medial collateral ligament (MCL) outside of the capsule. At 1, 2, 3, 4, 5 and 6 weeks after surgery, eight knees from each group were harvested. The joint gap was measured on posteroanterior radiographs after MCL-transection (MCLT). Gross examination and histological analysis were performed to evaluate cartilage damage to the medial femoral condyles, and knee joints were scanned using a Micro-CT system. The MCLT group experienced early stage OA from 3 to 6 weeks according to the histological scores. IL-6, MMP-1 and MMP-13 content in the synovial fluid were higher after MCLT than anterior cruciate ligament transection (ACLT) at 1 and 2 weeks.

A variety of genetic and environmental factors contribute to the progressive development of osteoarthritis (OA), such as synovial inflammation, obesity, age, trauma, genetics and joint instability^1^,^2^,^3^. However, little is known about the molecular mechanisms involved in the early stages of OA. This is because of our limited ability to detect early OA prior to the onset of pain, which only manifests during its late stages. *In vivo* experimental OA models are often used to study the kinetics of the progression of the disease in the whole animal. Reproducing the features of OA in animal models is crucial to gain a better understanding of disease progress, and to assess the benefits of potential therapies.

Animal OA models include naturally occurring OA during aging, transgenic models and surgically or chemically induced OA. Although spontaneously occurring OA models can follow the development of OA from its early stages, they are costly, time consuming and present with more variability in disease phenotype. The rabbit anterior cruciate ligament transection (ACLT) model is increasingly being used in early OA studies, and induces rapid and severe changes in articular cartilage and subchondral bone. However, ACLT models require the opening of the joint cavity which will inevitably affect gait and the initial oxygen tension and content of synovial fluid. It also caused rapid and severe progression of articular cartilage degeneration in ACLT models. This makes it difficult to capture the detailed information of the biologic changes during early stage OA. In this study we hypothesized that transecting the medial collateral ligament (MCLT) outside of the joint capsule could create a model of early osteoarthritis which exhibits gradual cartilage degeneration.

## Results

### Medial joint gap

**Figure 1:**
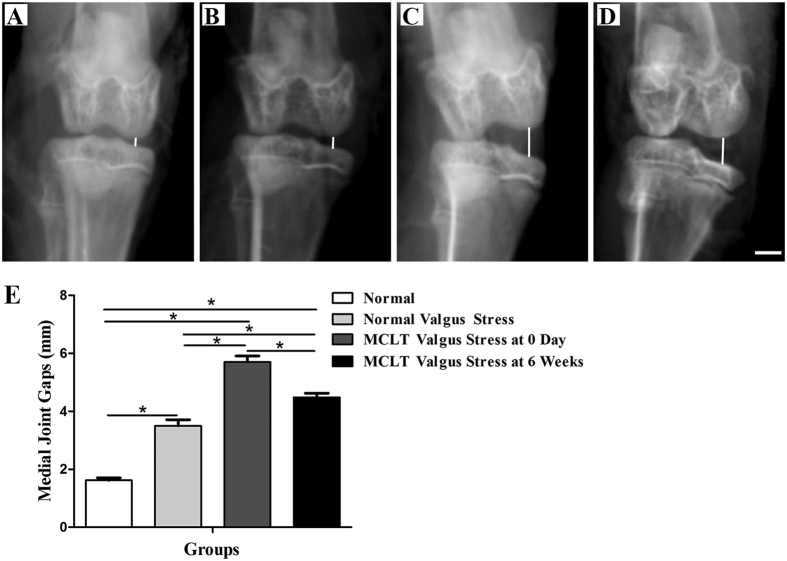
Medial joint gap measurements on the posteroanterior radiographs (**A**) normal distance of joint medial gap without valgus stress (**B**) normal distance of joint medial gap with valgus stress (**C**) distance of joint medial gap measured immediately after MCLT with valgus stress (**D**) distance of joint medial gap measured 6 weeks after MCLT with valgus stress (n = 6, scale bar, 0.5 cm) (**E**) Distance of joint medial gap measured immediately after MCLT with valgus stress increased significantly compared with normal distance of joint medial gap with valgus stress (P < 0.001). 6 weeks after the MCL-transection, the joint gap under valgus stress was still significantly wider than the uninjured knees (P = 0.005, n = 6, *p < 0.05).

The medial joint gap distance of normal rabbits without valgus stress was 1.62 ± 0.19 mm (n = 6). It increased to 3.5 ± 0.47 mm (n = 6) with valgus stress. The gap was measured immediately after MCLT (5.7 ± 0.46 mm, n = 6) and 6 weeks after MCLT (4.48 ± 0.32 mm, n = 6). The medial joint gap under valgus stress increased significantly after MCL-transection (n = 6, p < 0.001). Even though the macroscopic MCL healed 6 weeks after the operation, the joint gap under valgus stress was still significantly wider than the uninjured knees (n = 6, p = 0.005) (Fig. 1, Supplementary Table S1).

### Macroscopy

**Figure 2:**
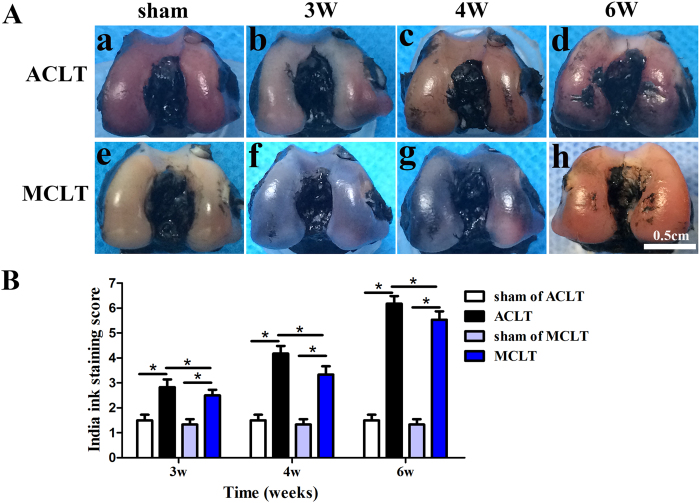
Macroscopic changes of articular cartilage (**A**) India ink staining of joints 3, 4 and 6 weeks after ACLT (b to d) and MCLT (f to h). India ink staining of sham groups (a,e). (n = 3, scale bar, 0.5 cm). (**B**) India ink staining score of knees. The India ink staining score of MCLT group was lower than that of ACLT group at the same time point (3 w, P = 0.006; 4 W, P = 0.044, 6 W, P = 0.044). (n = 8, *p < 0.05).

Damage to the medial femoral condyle after ACLT or MCLT was observed at 1, 2, 3, 4, 5 and 6 weeks after the procedure. Gross observations revealed that the surface of the cartilage was smooth in the ACLT and MCLT groups as well as their corresponding sham groups 1 and 2 weeks after the operation. India ink staining were performed at 3, 4 and 6 weeks after surgery. The India ink staining shows that the cartilage surface of sham groups was intact without retaining India ink. At 3, 4, and 6 weeks after ACLT or MCLT, the fibrillation of the cartilage surface appeared and became more obvious with time development. The India ink staining score of MCLT group was lower than that of ACLT group at the same time point (3 w, P = 0.006; 4 W, P = 0.044, 6 W, P = 0.044. Fig. 2A,B, Supplementary Table S2).

### MRI

**Figure 3:**
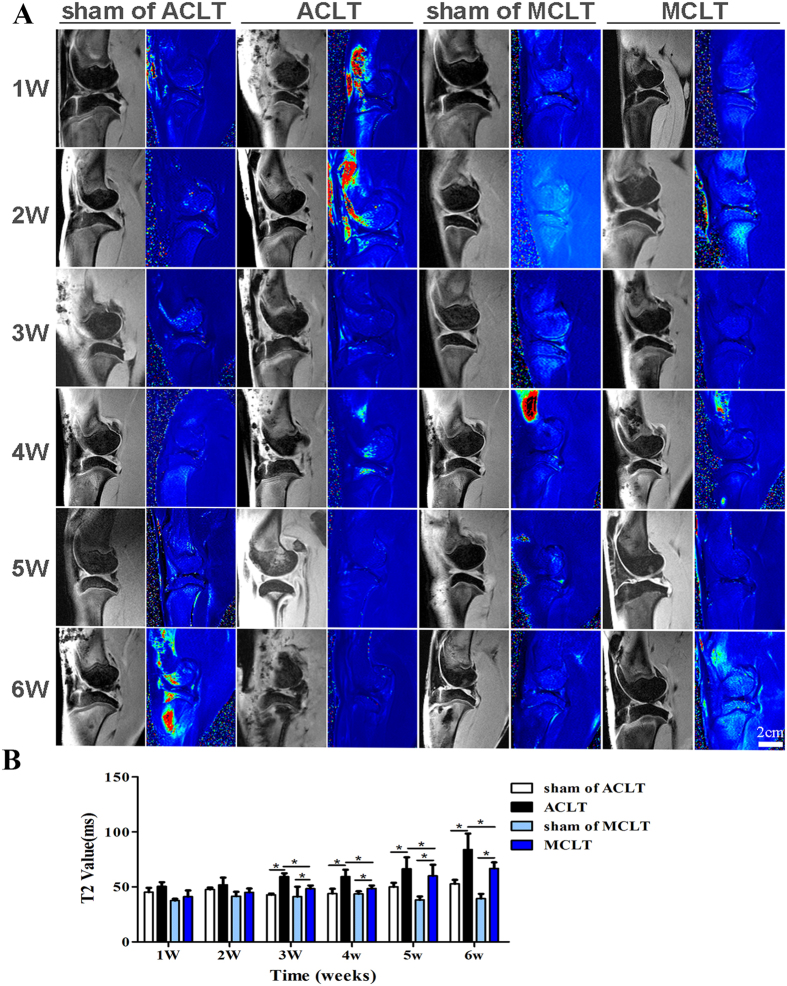
MRI examination and quantification of the knees (**A**) MR imaging and representative T2 mapping images of the knees at various time points (n = 3, scale bar, 2 cm). The black-white images are conventional MR images. And the colorful ones are T2 mapping images. The region within the white marking in T2-mapping image of the 1 W sham of ACLT represents the region of investigation. (**B**) Quantitative evaluation T2 relaxation time: The values in ACLT and MCLT groups have no significant difference with their corresponding sham groups at 1 and 2 weeks after surgical operation. The values of both ACLT and MCLT groups were higher than that of their corresponding sham groups at 3 weeks, and increased gradually over time. The values of MCLT group were significantly lower than those of ACLT group after 3 weeks. ((3 W, P = 0.001; 4 W, P = 0.008; 5 W, P = 0.031; 6 W, P = 0.039). (n = 3, *p < 0.05).

Conventional MRI allows for macroscopic anatomic assessment of the cartilage but is less sensitive to the biochemical changes associated with early OA. T2 mapping has demonstrated sensitivity to water content and collagen content as well as the structure and organization of cartilage^4^. We therefore used T2 mapping to quantitatively evaluate cartilage degeneration 1 to 6 weeks after surgery. Compared with their sham groups, both the MCLT and ACLT groups showed statistically significant differences in T2 mapping values from 3 to 6 weeks after surgery (Fig. 3A). The T2 mapping values of the MCLT group were significantly lower than those of the ACLT group (3 W, P = 0.001; 4 W, P = 0.008; 5 W, P = 0.031; 6 W, P = 0.039. Fig. 3B, Supplementary Table S3).

### SEM

**Figure 4:**
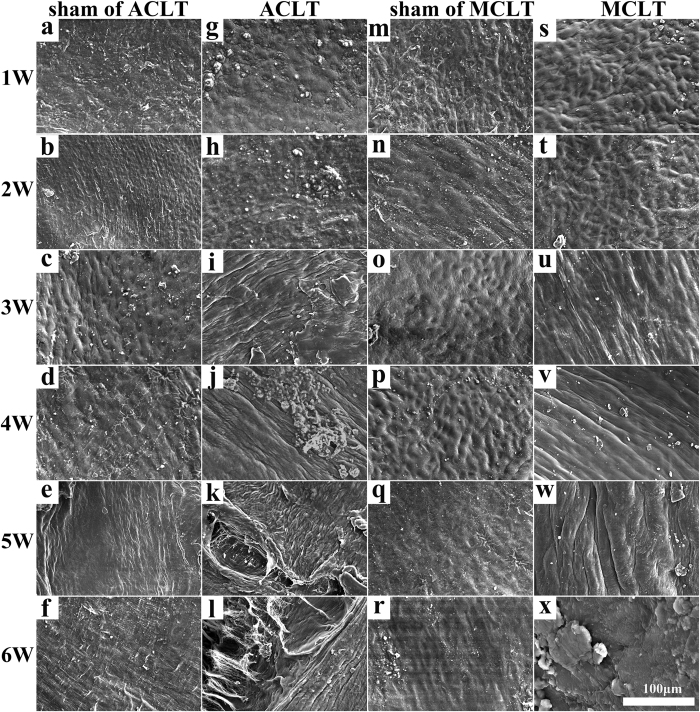
SEM imaging of the surface of the cartilage of the medial chondyle from 1–6 weeks after operation. There’s no obvious change in the surface of the cartilage of sham of ACLT group (**a**–**f**) and sham of MCLT group (**m**–**r**). In ACLT group (**g**–**l**), uneven and slightly rough surfaces were observed at 3 weeks. In MCLT group (**s**–**x**). a slightly uneven surface was observed at 3 and 4 weeks. The progression of cartilage degeneration in MCLT group was much slower than that of ACLT group. (n = 3, scale bar, 100 μm).

We investigated the ultrastructural changes of the cartilage surface after surgery using SEM. As shown in Fig. 4, sham ACLT and MCLT groups showed collagen fibrous structures on the cartilage surface, and the cartilage surface was even and smooth throughout the experimental period (Fig. 4). In the ACLT groups, uneven and slightly rough surfaces were observed 3 weeks after surgery (Fig. 4). Hillocky surfaces comprised of fibrous structures were observed at 4 weeks (Fig. 4), and leafy and split surfaces were observed 5 and 6 weeks post-ACLT (Fig. 4). In the MCLT group, a slightly uneven surface was observed 3 and 4 weeks after surgery. Rough and knobby surfaces were observed at 6 weeks, without a leafy split appearance (Fig. 4).

### Nanoindentation

**Figure 5:**
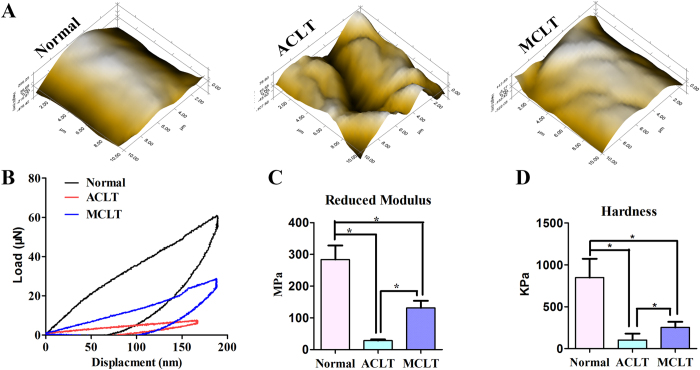
Biomechanical tests of cartilage. (**A**) Microscopic geomorphology of the carliage was acquired during nanoindentation. (**B**) Load-displacement curves of different groups were recorded within a test range of 400 nm. (**C**,**D**) The biomechanical properties of cartilage were calculated with the biomechanical curves: (**C**) Reduced modulus. The cartilage of the MCLT groups had a significantly higher reduced modulus value than the ACLT controls, but lower than normal (P values, normal : ACLT = 0.011, normal : MCLT = 0.039, ACLT : MCLT = 0.011) (**D**) Hardness. The cartilage of the MCLT group was harder than that of the ACLT controls but lower than normal knees (P values, normal : ACLT = 0.004, normal : MCLT = 0.013, ACLT : MCLT = 0.001.) (n = 3, *p < 0.05).

Six weeks post-surgery we performed biomechanical testing of the medial condyle cartilage with nanoindentation (Fig. 5). Using a micro-scanner, the articular surfaces in the ACLT group were scraggier and rougher than those of the MCLT and normal cartilage groups (Fig. 5A, Supplementary Table S10). The cartilage surfaces in the MCLT group were rougher than normal surfaces. According to load-displacement curves (Fig. 5B), we quantified the biomechanical properties of different groups. The cartilage of the MCLT groups had a significantly higher reduced modulus value than the ACLT controls, but lower than normal (P values, normal : ACLT = 0.011, normal : MCLT = 0.039, ACLT : MCLT = 0.011. Fig. 5C, Supplementary Table S4). Additionally, the cartilage of the MCLT group was harder than that of the ACLT controls but lower than normal knees (P values, normal : ACLT = 0.004, normal : MCLT = 0.013, ACLT : MCLT = 0.001. Fig. 5D, Supplementary Table S4).

### Synovial fluid

**Figure 6:**
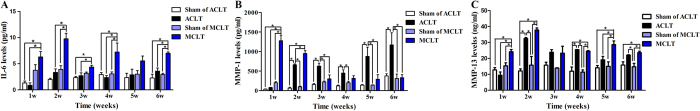
ELISA results of synovial fluid analysis (**A**) ELISA results of IL-6. The IL-6 level in the MCLT group was higher than in the ACLT group from 1 to 6 weeks after surgery, especially at 1 and 2 weeks (P values, 1 W, P < 0.001; 2 W, P < 0.001; 3 W, P = 0.032; 4 W, P < 0.001; 5 W, P = 0.001; 6 W, P < 0.001). (**B**) ELISA results of MMP-1. The MMP-1 level was higher in the MCLT compared with the ACLT group 1 and 2 weeks post-surgery, although the ACLT group levels were higher from 3 to 6 weeks (P values, 1 W, P < 0.001; 2 W, P = 0.009; 3 W, P = 0.004; 4 W, P = 0.194; 5 W, P < 0.001; 6 W, P < 0.001). (**C**) ELISA results of MMP-13. The MMP-13 levels in the MCLT groups were higher than in the ACLT group 1 week after the operation (P values. 1 W, P = 0.02). (n = 8, *p < 0.05).

The IL-6 level in the MCLT group was higher than in the ACLT group from 1 to 6 weeks after surgery, especially at 1 and 2 weeks (P values, 1W, P < 0.001; 2 W, P < 0.001; 3 W, P = 0.032; 4 W, P < 0.001; 5 W, P = 0.001; 6 W, P < 0.001; Fig. 6A). The MMP-1 level was higher in the MCLT compared with the ACLT group 1 and 2 weeks post-surgery, although the ACLT group levels were higher from 3 to 6 weeks (P values, 1 W, P < 0.001; 2 W, P = 0.009; 3 W, P = 0.004; 4 W, P = 0.194; 5 W, P < 0.001; 6 W, P < 0.001; Fig. 6B). Meanwhile, the MMP-13 levels in the MCLT groups were higher than in the ACLT group 1 week after the operation, though the levels trended lower at 3 and 4 weeks (P values, 1 W, P = 0.02; Fig. 6B).

### Histology

**Figure 7:**
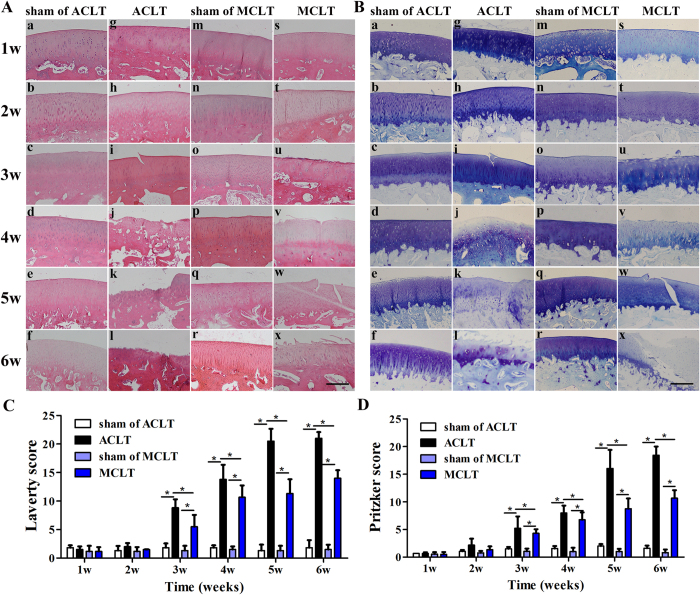
Histological assessment and microscopic observation of the cartilage of the medial chondyle at 1, 2, 3, 4, 5 and 6 weeks after the procedure (**A**) Hematoxylin & eosin staining of samples. (a–f) show the sham of ACLT groups’ cartilage degeneration. (g–l) show the ACLT groups’. (m–r) show the sham of MCLT groups’. (s–x) show the MCLT groups’. (n = 8, scale bar, 100 μm). (**B**) Toluidine blue staining of of samples. (a–f) show the sham of ACLT groups’ cartilage degeneration. (g–l) show the ACLT groups’. (m–r) show the sham of MCLT groups’. (s–x) show the MCLT groups’. (n = 8, scale bar, 100 μm) (**C**) Laverty score for samples. The scores of the MCLT group were significantly lower than the ACLT group from 3 to 6 weeks after surgery (3 W, P < 0.001; 4 W, P = 0.04; 5 W, P < 0.001; 6 W, P < 0.001). (**D**) Pritzker score for samples. The scores of the MCLT group were significantly lower than the ACLT group from 3 to 6 weeks after surgery (3 W, P = 0.002; 4 W, P = 0.038; 5 W, P = 0.001; Pritzker score, P < 0.001). (n = 8, *p < 0.05).

Histological changes to the cartilage surface were detected using HE and toluidine blue staining. HE and toluidine staining showed no apparent histologic degeneration in both sham groups throughout the experimental period (Fig. 7). The surface of the cartilage in the ACLT group was intact 1 week after surgery, and uneven and slightly rough at 2 weeks. A slightly split surface was observed at 3 weeks and the toluidine blue staining became lighter than the sham group at this time point. Some complex vertical fissures were found with matrix loss at 4 weeks, and mid-zone excavation and cyst formation within the cartilage matrix could be seen 5 and 6 weeks after surgery. In the MCLT group, the surfaces of the cartilage were intact at 1 and 2 weeks, and became slightly rough at 3 weeks. Some vertical fissures were detected at 4 and 5 weeks, and the fissures increased and extended into deeper layers at 6 weeks (Fig. 7A,B). The toluidine blue staining became lighter than the sham group from 3 weeks.

We used the Osteoarthritis Research Society International (OARSI) recommended Laverty and Pritzker osteoarthritis cartilage histopathology assessment systems to evaluate early cartilage degeneration (Supplementary Tables S5 and S6). As shown in Fig. 7C,D, compared with their sham group, the ACLT and MCLT group showed no significant difference in Laverty and Pritzker score 1 to 2 weeks after surgery. After 3 weeks, the Laverty and Pritzker scores were increased in both groups compared with their corresponding sham group (Fig. 7A). The scores of the MCLT group were significantly lower than the ACLT group from 3 to 6 weeks after surgery (MCLT : ACLT, 3 W, Laverty score, P < 0.001, Pritzker score, P = 0.002; 4 W, Laverty score, P = 0.04, Pritzker score, P = 0.038; 5 W, Laverty score, P < 0.001, Pritzker score, P = 0.001; 6 W, Laverty score, P < 0.001, Pritzker score, P < 0.001).

### Immunohistochemistry

**Figure 8:**
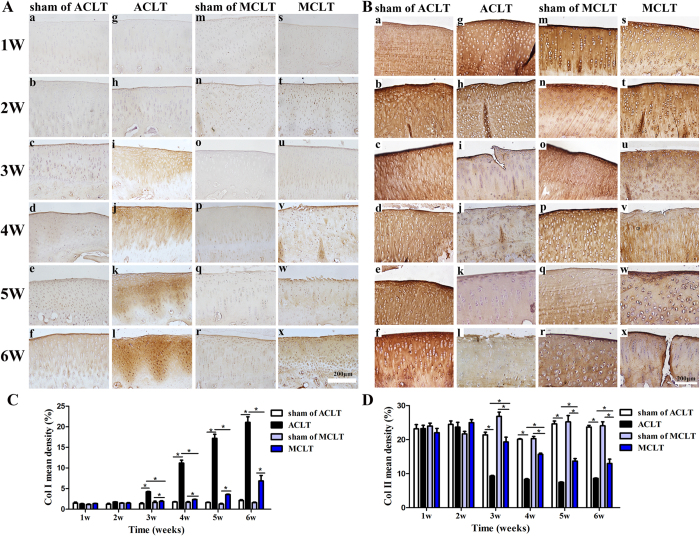
Immunohistochemistry staining of type I and II collagen of the medial chondyle cartilage at 1, 2, 3, 4, 5 and 6 weeks after the procedure. (**A**) Type I collagen changes. (a–f) show the sham of ACLT groups’ cartilage degeneration. (g–l) show the ACLT groups’. (m–r) show the sham of MCLT groups’. (s–x) show the MCLT groups’. (n = 8, scale bar, 200 μm). (**B**) Type II collagen changes. (a–f) show the sham of ACLT groups’ cartilage degeneration. (g–l) show the ACLT groups’. (m–r) show the sham of MCLT groups’. (s–x) show the MCLT groups’. (n = 8, scale bar, 200 μm). (**C**) Mean collagen I density. MCLT group was lower than the ACLT group from 3 to 6 weeks (MCLT : ACLT, 3 W, P < 0.001; 4 W, P = 0.001; 5 W, P < 0.001; 6 W, P < 0.001). (**D**) Mean collagen II density. MCLT group was lower than the ACLT group from 3 to 6 weeks. (MCLT : ACLT, 3 W, P = 0.001; 4 W, P < 0.001; 5 W, P < 0.001; 6 W, P = 0.009) (n = 8, *P < 0.05).

Immunohistochemistry for type I, II collagen was used to assess cartilage degeneration (Fig. 8, Supplementary Tables S7 and S8). After 3 weeks, the mean density of type I collagen were significantly increased in both ACLT and MCLT groups compared with their corresponding sham groups. The mean collagen I density of MCLT group was lower than the ACLT group from 3 to 6 weeks (MCLT : ACLT, 3 W, P < 0.001; 4 W, P = 0.001; 5 W, P < 0.001; 6 W, P < 0.001). The mean density of type II collagen were significantly decreased in both ACLT and MCLT groups compared with their corresponding sham groups after 3 weeks. The mean density of MCLT group was higher than the ACLT group from 3 to 6 weeks (MCLT : ACLT, 3 W, P = 0.001; 4 W, P < 0.001; 5 W, P < 0.001; 6 W, P = 0.009).

### Subchondral bone

**Figure 9:**
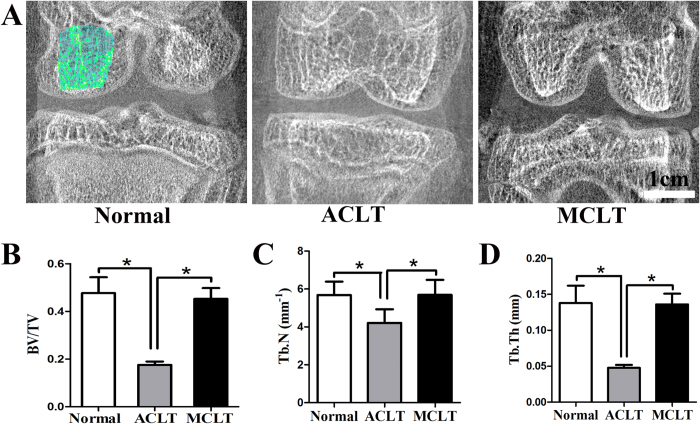
Subchondral bone changes of medial chondyle (**A**) representative 2D reconstructed coronal μCT images of subchondral bone for normal joints, ACLT and MCLT group at 6 weeks. (n = 5, scale bar, 1 cm). The green area shows the representative region of interest (ROI). (**B**) quantitative analysis of bone volume/total volume (BV/TV, P < 0.001) (**C**) quantitative analysis of trabecular number (Tb. N, P = 0.008) (**D**) quantitative analysis of trabecular thickness (Tb. Th, P = 0.008) (n = 5, *p < 0.05).

Subchondral bone changes were analyzed in the medial condyle region 6 weeks after surgery using micro-CT images (Fig. 9, Supplementary Table S9). Quantitative analysis revealed that bone volume/total volume (BV/TV, Fig. 9B), trabecular number (Tb. N, Fig. 9C) and trabecular thickness (Tb. Th, Fig. 9D) of MCLT group didn’t decreased compared with the normal ones’. Those of ACLT group decreased significantly compared with those of the MCLT group (BV/TV, P < 0.001; Tb. Th, P = 0.008; Tb. N, P = 0.008; n = 3).

## Discussion

Various animal models have been used to evaluate the pathophysiology and treatment outcomes of OA, including surgical and injection OA induction methods^1^. Surgically-induced OA models are more representative of posttraumatic changes^5^. Different animals have been used in an induced OA model, such as mice, rats^6^, Guinea pigs^7^, rabbits^8^, dogs^9^, sheep^10^, horses^11^ and mini pigs^4^. Surgical methods include anterior cruciate ligament transection (ACLT), media meniscal tear (MMT), partial meniscectomy, medial meniscectomy, lateral meniscectomy, ACLT/MCLT combined with MMT, ACLT/MMT and MCLT/MMT. Any surgical procedures, even a sham procedure such as a capsular incision, will cause inflammation, pain and possibly altered gait 1. One common disadvantage of these surgically induced animal models is that they may affect the initial structures and content of synovial fluid in joint and progress too rapidly to end-stage degeneration. To study the early changes of OA in detail, especially the changes of synovial fluid and cartilage, we need to create an animal model of early stage OA without interrupting the initial structures and content of synovial fluid in joint at the time of induction.

Though many studies have discussed the injury and healing of the MCL, no article discussed the importance of protecting the joint capsule or compared cartilage changes related to this technique in detail^12^. Roberts *et al.* as well as Bove *et al.* performed MCLT in combination with meniscal transection and observed OA induction^13^,^14^. But they didn’t report the cartilage situation while doing the MCL-transection alone. And doing meniscal transection would open the joint capsule. Allen *et al.* reported the transection of the medial collateral ligament and medial meniscus of male Lewis rats resulted in changes in hind paw weight bearing and the development of tactile allodynia (secondary hyperalgesia). In their study, they transected the medial collateral ligament and visualized the joint space as a MCL sham control group. They reported no changes were noted to the cartilage at 3 weeks after operation^15^. But they opened the capsule when doing MCLT which induced the loss of cartilage degrading enzymes (e.g. MMP -1, MMP-13 etc.). The intact cartialge in thier research may caused by the loss of degrading enzymes. And the species differences between the rabbit and rat may lead to the different cartilage changes. In this study, we created an animal model of early cartilage degeneration by resecting the MCL without injuring the joint capsule, and compared its features with the commonly used ACLT model. The results of medial joint gap distance indicate that transection of the MCL outside of the capsule led to the instability of the joint. This instability partially persisted 6 weeks after MCLT. Results of gross observations, histology, SEM and MRI images all suggest that damage to articular cartilage emerged 3 weeks after the original surgical procedure and gradually increased over time in both the ACLT and MCLT groups. Cartilage degeneration in the MCLT group was less severe and much slower than in the ACLT group. The results of nanoindentation identified the slower biomechanical changes in the MCLT group than the ACLT group. Pritzker scores confirmed that the cartilage injury in the MCLT group was early stage OA^16^. Compared with sham groups, toluidine blue staining of samples became lighter both in ACLT and MCLT groups from 3 weeks after operation. And the staining of MCLT group was more deeper than ACLT group from 4 to 6 weeks which supported the progression of cartilage degeneration in the MCLT group was much slower than in the ACLT group. The results of micro-ct measurements indicate that subchondral trabecular bone after extracapsular MCL-transection changed slower than ACL-transection.

The main advantage of this model is that transecting the MCL outside the joint capsule will not interrupt the initial structures and content of synovial fluid in joint, which suggests that it is more suitable for detecting synovial fluid information of early OA cartilage degeneration. Surgical procedures that require arthrotomies can result in the primary loss of synovial fluid and may affect the metabolism of the cartilage. In our experiments, the IL-6, MMP-1 and MMP-13 ELISA results in the MCLT group were higher than in the ACLT group at 1 and 2 weeks after surgery. These measurements indicate that extracapsular MCL-transection provided more synovial fluid information than ACL-transection, especially 1 and 2 weeks after the operation. These information could be associated with early cartilage degeneration. C.O. Tibesku^17^ also reported that the healing of the joint injury increased the expression of VEGF in the sham ACLT group^18^. With the capsule incised, the cartilage was exposed to the air which might affect the chondrocytes with an unknown mechanism of action. Low oxygen tension (5%), which might be considered hypoxic for other cell types but is normal for articular cartilage, was reported to promote a chondrocyte phenotype^2^. The normal air environment may add no benefit to the chondrocytes. Furthermore, loss of the primary synovial fluid may injure the cartilage and ion (Na^+^/K^+^ pump activity, Na^+^/Ca2^+^) homeostasis may be disturbed^2^. Although the synovial fluid may return to normal 2 to 3 weeks after surgery, information obtained before 2 or 3 weeks after the procedure may be altered. Moreover, synovial inflammation begins immediately after the operation^19^. Injection-induced OA models that stimulate intra-articular inflammation, chondrocyte toxicity and matrix damage may also change the initial content of synovial fluid in joint. No matter what is injected into the joint, the components will be changed. There is no doubt that it will influence the measurements of the synovial fluid of early stage OA. van Vulpen^17^ has recently reported IL-1β, in contrast to TNFα, is pivotal in blood-induced cartilage damage. Our animal model could successfully avoid the disadvantages of opening the capsule.

Another advantage of our model is that the progression of OA is slower than in the traditional ACLT model^20^. Based on the results of the pathology and SEM images of the cartilage, early degeneration started at the third week after surgery, with the cartilage surface becoming slightly rough. From the third week after surgery, surface abrasions could be found. In the ACLT model and some other surgically-induced OA models, more severe damage can be detected^20^ that may make it difficult to capture more detailed information about early OA. For example, the level of a disintegrin and metalloproteinase with thrombospondin motifs-4 (ADAMTS-4) increases rapidly during early stage OA, and then may decrease during the middle and late stages^21^.

## Conclusion

We have characterized a novel knee injury rabbit model to study early stage OA. This model holds significant advantages over other models of OA, as it induces a slight MCL injury that is outside of the joint capsule without interrupting initial structures and content of synovial fluid in joint. The most important advantage of this model is that it will be more suitable for synovial fluid research during the early stage of OA. This model is easy to implement, highly reproducible and a promising tool for early diagnosis and treatment studies.

## Materials and Methods

### Study design and surgery

**Figure 10:**
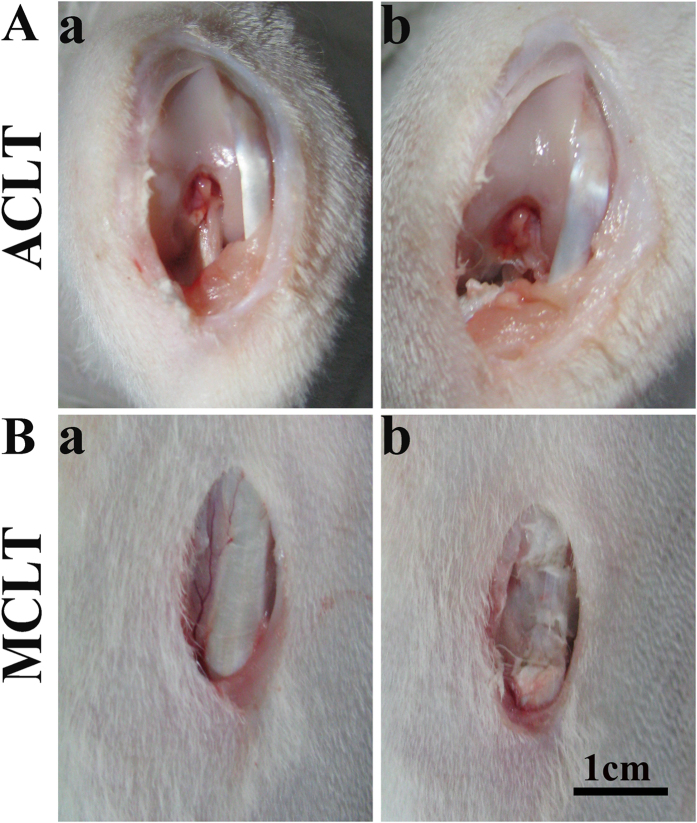
Macroscopic review of ACLT and MCLT (**A**) steps of ACLT (a) showed the morphology of ACL. (b) showed the ACL was fully transected. (**B**) steps of transecting MCL outside of the joint capsule (a) showed the morphology of MCL. (b) showed the bottom dead center of the medial collateral ligament was transected. The distal 3–4 mm part of the MCL was resected without damage to the joint capsule. (Scale bar, 1 cm).

New Zealand white rabbits (n = 96, age 4–6 months, weight 2.5–3.0 kg) were divided into 4 groups (ACLT, sham of ACLT, MCLT or sham of MCLT). Eight knees in every group were harvested 1, 2, 3, 4, 5 and 6 weeks after surgery. After anesthesia and routine preparation, the left knee was either an operated ACLT or a sham ACLT, while the right knee (contralateral joint) was either an operated MCLT or a sham MCLT. The ACLT procedure was performed in a previously reported manner^22^ (Fig. 10A). The MCLT was performed as follows (Fig. 10B): a medial incision was made over the knee 2 cm from the tibial tubercle. The lower attachment of the medial collateral ligament was transected and the distal 3–4 mm of the MCL was carefully resected without damaging the joint capsule. To verify knee instability, a valgus stress test in 0° and 30° of flexion was performed intraoperatively. Postoperatively, all animals were allowed free movement in their own boxes with daily check-ups for signs of wound healing problems or infections. Eight rabbits in each group were euthanized with overdose of anesthesia 1, 2, 3, 4, 5 and 6 weeks after the procedure, and the femoral condyles of both knees were harvested. There was no difference in weight gain between the rabbit groups throughout the experiment. All animal experimental protocols were approved by the Animal Care and Use Committee of Peking University and were in compliance with the Guide for the Care and Use of Laboratory Animals (National Academies Press, National Institutes of Health Publication No. 85-23, revised 1996). The methods were carried out in accordance with the approved guidelines.

### Posteroanterior radiograph

The joint gap from the middle site of medial femoral to middle site of medial tibia plateau was measured on posteroanterior radiographs after MCL-resection as a verification of joint instability. The normal medial knee joint gaps and the normal joint gaps under valgus stress were measured as controls (n = 6). After extracapsular MCL-resection, the medial joint gaps under valgus stress were measured immediately (n = 6). The gaps were measured again 6 weeks after MCL-resection (n = 6). Each sample was measured three times by two observers.

### MRI

At 1, 2, 3, 4, 5 and 6 weeks after the operation, rabbits were sacrificed and frozen in −80° before performed magnetic resonance imaging (MRI) for evidence of early cartilage injury using a Siemens TIM Trio 3 T (T) MRI scanner (Siemens, Erlangen, Germany) with a small animal-specific knee coil (Chenguang Medical Technologies Co., Ltd., Shanghai, China) to improve the signal-to-noise and contrast-to-noise ratios. The protocol included five sequences (T1, T2, T1-Flash 3D, PD, T2-map, Supplementary Table S10) for morphologic observation and quantitative analysis. The total acquisition time was approximately 40 min. T2-mapping values of medial chondyle were analyzed by a senior musculoskeletal radiologist using an inline processing package (SyngoMapIt; Siemens), as previously described^23^,^24^.

### Macroscopy

Gross examination of each knee was performed to evaluate for cartilage injury and surface smoothness using a macroscopic score system^25^. India ink staining was performed on samples from the sham group, 3, 4, and 6 weeks after the operation (n = 8). And the India ink staining score was evaluated using the score system recommended by OARSI which is semiquantitative with 1 point for intact cartilage surface and 7 points for more than 5 mm full depth erosion^26^.

### SEM

High-resolution scanning electron microscopy (SEM) was used to assess microscopic changes in the surfaces of the the middle loading area of the medial femoral condyles. Briefly, samples (n = 3) of the medial condyles were trimmed without disturbing the cartilage surface, fixed immediately in 4 mL of 25% glutaraldehyde for 1 day at 4 °C, dehydrated in a graded ethanol series, and subjected to critical point drying to ensure complete dehydration. The surfaces of the knees were coated with a 5-nm layer of gold in a high-vacuum gold spatter coater, and then viewed using a high-resolution SEM (S-2500; Hitachi Ltd., Tokyo, Japan).

### Nanoindentation

Biomechanical analysis of repaired tissues was performed using nanoindentation^27^. Samples (n = 3) were isolated from the load-bearing area of the medial condyle. All indentations were performed using the TriboIndenter (Hysitron Inc., Minneapolis, MN, USA) with a 400-mm radius curvature conospherical diamond probe tip. A trapezoidal load function was applied to each indent site with loading (10 s), hold (2 s), and unloading (10 s). Indentations were force-controlled to a maximum indentation depth of 200 nm. Meanwhile, the microscopic geomorphology of the indentation zones was captured using micro-scanning apparatus.

### Histology

At 1, 2, 3, 4, 5 and 6 weeks after the operation, the injured knees (n = 8) were harvested, fixed in 4% paraformaldehyde (pH 7.4) for 48 h at 4 °C and decalcified in 15% EDTA (pH 7.2 in PBS) with 5% paraformaldehyde at 4 °C. The decalcified medial condyles were trimmed, dehydrated in a graded ethanol series and embedded in paraffin. Serial sagittal sections (5 μm thick) of the middle loading area of the medial femoral condyle were cut and stained with hematoxylin & eosin (HE) and toluidine blue according to the standard protocals. The toluidine blue-stained sections were evaluated using the OARSI score established by Laverty^26^ and Pritzker *et al.*^16^. The sections were examined by three investigators blinded to the treatment groups.

Immunohistochemistry was performed using antibodies against type I collagen (Calbiochem Cat No. ab90395; Cambridge, MA, USA) and type II collagen (Calbiochem Cat No. CP18-100UG; Novabiochem, Boston, MA, USA) according to standard protocols.

### Microcomputed tomography

Six weeks after the operation, the injured knees (n = 5) were imaged using a μCTscanner (Inveon, Siemens Medical Solutions USA, Inc., IL, USA) at a 36-μm spatial resolution, 80-kV voltage, 500-μA current, 900-ms integration time, and 360 projections per 360°. A cylindrical volume of interest (VOI) 5 mm in diameter and 5 mm in height was defined as the subchondral spongiosa region of medial chondyle of each knee for evaluation. Bone volume/total volume (BV/TV), trabecular number (Tb. N), trabecular thickness (Tb. Th), and trabecular separation (Tb. Sp) were calculated. The same technician blinded to animal treatment assignment conducted the microcomputed tomography (μCT) analyses.

### Synovial fluid

At 1, 2, 3, 4, 5 and 6 weeks after the operation, the joints were washed by injecting 1500 μl of normal saline into the knee joint capsule. The synovial fluid was then aspirated from the knees using a sterile knee puncture just prior to the harvesting procedure. The specimen was then centrifuged to remove cells and stored at −80 °C for further enzyme-linked immunosorbent assay (ELISA) measurements. According to the manufacturer’s instructions, synovial fluid interleukin-6 (IL-6), matrixmetalloproteinase-1 (MMP-1) and matrixmetalloproteinase-13 (MMP-13) levels were measured using a commercial sandwich ELISA development kit (R&D Systems, Minneapolis, MN, USA).

### Statistics

All data are expressed as mean ± standard deviation. All experiments were repeated at least three times. After testing for homogeneity of variances, a one-way analysis of variance (ANOVA) was used to evaluate differences between groups. Pairwise post hoc tests were performed with Fisher’s LSD multiple comparisons test. P-values < 0.05 were considered significant.

## Additional Information

**How to cite this article**: Liu, Z. *et al.* A novel rabbit model of early osteoarthritis exhibits gradual cartilage degeneration after medial collateral ligament transection outside the joint capsule. *Sci. Rep.* **6**, 34423; doi: 10.1038/srep34423 (2016).

## Supplementary Material

Supplementary Information
