# Supplementary material for: A novel rabbit model of early osteoarthritis exhibits gradual cartilage degeneration after medial collateral ligament transection outside the joint capsule
Source: Sci Rep. 2016 Oct 19;6:34423. doi: 10.1038/srep34423 (PMC5069470; doi:10.1038/srep34423)
Supplement: Supplementary Information [file srep34423-s1.pdf]

A novel rabbit model of early osteoarthritis exhibits gradual cartilage degeneration after medial collateral ligament transection outside the joint capsule

Zhenlong Liu*[1], Xiaoqing Hu*[1], Zhentao Man[1], Jiying Zhang[1], Yanfang Jiang[1], Yingfang Ao** [1]

[1] *Institute of Sports Medicine, Beijing Key Laboratory of Sports Injuries, Peking University Third Hospital, 49 North Garden Road, Haidian District, Beijing 100191, People's Republic of China*

Correspondence and requests for materials should be addressed to Y.A. (email: aoyingfang@163.com)

Supplementary Table S1 Descriptive statistics for medial joint gap (mean±SD，n=6)

	normal (N)	normal valgus stress (NVS)	MCLT valgus stress at 0 day (MVS0)	MCLT valgus stress at 6 weeks (NVS6)
medial joint gap (mm)	1.62±0.19	3.5±0.47	5.7±0.46	4.48±0.32
P values	N:NVS<0.001, NVS:MVS0<0.001, NVS:MVS6=0.005, MVS0:MVS6=0.001			

Supplementary Table S2 Descriptive statistics for India ink staining score at 3，4 and 6 weeks after sugery (mean±SD，n=6)

	3w	4w	6w
sham of ACLT	0.67±0.29	0.72±0.25	0.67±0.30
ACLT	0.67±0.21	0.94±0.39	0.83±0.18
sham of MCLT	0.83±0.35	2.33±0.42	0.88±0.17
MCLT	0.72±0.25	2.4±0.34	0.72±0.14

Supplementary Table S3 Descriptive statistics for T2 mapping values (ms) at 1，2，3，4，5 and 6 weeks after sugery (mean±SD，n=3)

	sham of ACLT	ACLT	sham of MCLT	MCLT
1W	45.16±4.06	50.42±3.96	37.62±1.55	41.2±5.61
2W	47.68±1.75	51.78±6.65	41.62±4.00	45±3.60
3W	41.14±9.06	59.4±3.06	38.9±3.83	48.44±2.82
4W	43.92±4.40	59.32±6.31	43.66±2.41	48.56±2.78
5W	50.04±3.79	72.44±5.08	38.16±3.21	60±10.21
6W	52.78±3.71	83.96±14.55	39.48±4.17	66.78±5.47

Supplementary Table S4 Descriptive statistics for nanoindentation at 6 weeks after sugery (mean±SD，n=3)

	reduced modulus (MPa)	hardness (KPa)
normal	283.9±99.27	847.60±224.94
ACLT	28.52±7.825	82.67±36.56
MCLT	131.4±33.16	292.85±29.90
P value	normal:ACLT=0.011<br>normal:MCLT=0.039<br>ACLT:MCLT=0.011	normal:ACLT=0.004<br>normal:MCLT=0.013<br>ACLT:MCLT =0.001

Supplementary Table S5 Descriptive statistics for Laverty score at 1，2，3，4，5 and 6 weeks after sugery (n = 6, P < 0.05)

group	Safranin o-fast green	structure	chondrocyte density	cluster formation	total score
1w sham of ACLT	0.83±0.41	0.67±0.52	0	0	1.83±0.41
1W ACLT	0.67±0.52	0.83±0.41	0	0	1.5±0.55
1W sham of MCLT	0.67±0.52	0.5±0.55	0	0	1.17±0.98
1W MCLT	0.83±0.41	0.33±0.52	0	0	1.17±0.75
2w sham of ACLT	0.83±0.41	0.5±0.55	0	0	1.33±0.81
2W ACLT	0.67±0.52	0.83±0.41	0.5±0.55	0	2±0.63
2w sham of MCLT	0.67±0.52	0.5±0.55	0	0	1.17±0.75
2W MCLT	0.83±0.41	0.5±0.55	0.17±0.41	0	1.5±1.05
3w sham of ACLT	0.83±0.41	0.83±0.41	0.17±0.41	0	1.83±0.75
3W ACLT	2.83±0.75	3.83±0.98	1.83±0.75	0.33±0.52	8.83±1.47
3w sham of MCLT	0.83±0.41	0.5±0.55	0	0	1.33±0.82
3W MCLT	1.83±0.75	2.83±0.75	0.5±0.55	0.33±0.52	5.5±2.07
4w sham of ACLT	0.5±0.55	0.83±0.41	0.33±0.52	0.33±0.52	1.83±0.41
4W ACLT	4±0.89	7.17±0.75	2.17±0.75	0.5±0.55	13.8±2.56
4w sham of MCLT	0.83±0.41	0.33±0.52	0.33±0.52	0	1.5±0.55
4W MCLT	4.17±0.75	4.5±1.05	1.5±0.55	0.5±0.55	10.67±2.07
5w sham of ACLT	1±0.63	0.33±0.52	0	0	1.33±1.03
5W ACLT	5.5±0.55	9.83±0.75	3.33±0.82	1.83±0.75	20.5±2.17
5w sham of MCLT	0.5±0.55	0.83±0.41	0.17±0.41	0	1.33±0.82
5W MCLT	3.83±0.75	6.17±0.75	0.83±0.75	0.5±0.55	11.33±2.50
6w sham of ACLT	0.83±0.41	0.5±0.55	0.33±0.55	0.17±0.41	1.83±1.33
6W ACLT	5.5±0.55	10±0.89	3.17±0.75	2.33±0.55	21±1.1
6w sham of MCLT	0.67±0.55	0.5±0.55	0.33±0.55	0	1.5±0.84
MCLT	4.5±0.84	7.17±0.75	1.5±0.55	0.83±0.75	14±1.41

Supplementary Table S6　Descriptive statistics for Pritzker score at 1，2，3，4，5 and 6 weeks after sugery (mean ±SD, n = 8, P < 0.05)

	**sham of ACLT**			**ACLT**			**sham of MCLT**			**MCLT**		
	**Stage**	**Grade**	**Stage× Grade**	**Stage**	**Grade**	**Stage× Grade**	**Stage**	**Grade**	**Stage× Grade**	**Stage**	**Grade**	**Stage× Grade**
**1W**	**0.72±0.14**	**0.72 ± 0.14**	**0.72±0.14**	**0.67 ± 0.21**	**0.67±0.21**	**0.67±0.21**	**0.56 ± 0.34**	**0.61 ± 0.39**	**0.56±0.35**	**0.499 ± 0.35**	**0.61 ± 0.39**	**0.50±0.35**
**2W**	**0.89±0.17**	**1.06 ± 0.25**	**1.06±0.25**	**1.22 ± 0.40**	**1.61±0.57**	**2.17±1.17**	**0.47 ± 0.21**	**0.83 ± 0.41**	**0.78±0.34**	**1.17±0.50**	**1.50 ± 0.62**	**1.95±1.42**

3W	1±0.21	1.39 ± 0.33	1.5±0.35	2.11 ± 0.17	3±0.47	6.44±1.11	0.78 ± 0.27	1.06 ± 0.49	1.06±0.49	1.56±0.34	2.39 ± 0.57	3.67±1.25
4W	1±0.21	1.5±0.35	1.56±0.46	2.17 ± 0.18	3.75±0.61	8±1.32	0.72 ± 0.49	0.94 ± 0.65	1.00±0.70	2.11±0.27	2.97 ± 0.92	6.95±2.00
5W	1±0.00	2.00 ± 0.37	2±0.37	3.33 ± 0.37	4.83±0.59	16.06±3.34	0.78 ± 0.27	1±0.42	1.00±0.52	2.28±0.14	4±0.6	9.00±1.52
6W	1±0.21	1.50 ± 0.35	1.61±0.49	3.67 ± 0.21	5.06±0.57	18.44±1.56	0.61 ± 0.39	0.83 ± 0.55	0.83±0.55	2.67±0.52	3.89 ± 0.69	9.89±1.69

Supplementary Table S7 Descriptive statistics for collagen I mean density (%) at 1，2，3，4，5 and 6 weeks after sugery (mean±SD，n=8)

	sham of ACLT	ACLT	sham of MCLT	MCLT
1W	1. 47±0. 55	1. 26±0. 3	1. 13±0. 24	1. 36±0. 09
2W	1. 19±0. 47	1. 73±0. 13	1. 46±0. 17	1. 45±0. 30
3W	1. 33±0. 47	4. 22±0. 24	1. 63±0. 73	1. 89±0. 33
4W	1. 76±0. 18	11. 2±1. 58	1. 65±0. 43	2. 36±0. 16
5W	1. 62±0. 22	17. 2±2. 17	1. 23±0. 39	3. 56±0. 21
6W	2. 11±0. 44	21. 08±3	1. 6±0. 27	6. 86±2. 93

Supplementary Table S8 Descriptive statistics for collagen II mean density (%) at 1，2，3，4，5 and 6 weeks after sugery (mean±SD，n=8)

	sham of ACLT	ACLT	sham of MCLT	MCLT
1W	23. 16±2. 82	23. 24±2. 17	23. 98±1. 93	22. 03±2. 80
2W	24. 47±2. 25	23. 67±3. 19	21. 66±1. 68	24. 99±1. 99
3W	21. 4±1. 78	9. 29±0. 42	26. 82±2. 84	19. 32±3. 1
4W	20. 1±0. 35	8. 3±0. 46	20. 3±1. 41	15. 66±0. 85
5W	24. 65±1. 8	7. 46±0. 23	25. 2±4. 15	13. 7±1. 65
6W	23. 69±1. 13	8. 6±0. 27	24. 07±2. 62	12. 99±2. 83

Supplementary Table S9 Descriptive statistics for micro - CT at 6 weeks after sugery (mean±SD，n=3)

	BV/TV (%)	Tb.N（mm^{-1}）	Tb.Th（mm）
normal（1）	47.7±6.7	5.682±0.709	0.138±0.054
ACLT（2）	17.6±1.4	4.209±0.720	0.048±0.009
MCLT（3）	45.3±4.5	5.690±0.791	0.136±0.333
P value	normal:ACLT <0.001	normal:ACLT =0.008	normal:ACLT =0.048
	normal:MCLT =0.436	normal:MCLT =0.987	normal:MCLT =1
	ACLT:MCLT <0.001	ACLT:MCLT =0.008	ACLT:MCLT =0.008

Supplementary Table S10 Concrete values of loading-displacement of nanoindentation					
Normal		ACLT		MCLT	
displacement(nm)	load(μ N)	displacement(nm)	load(μ N)	displacement(nm)	load(μ N)
-11.23541	-0.462633	-33.36116	-1.300043	-12.23815	-0.250632
-11.10585	-0.522059	-34.19515	-1.041283	-12.48384	-0.159626
-9.788238	-0.35563	-32.65033	-1.179814	-11.54097	-0.002826
-9.318839	-0.474477	-32.125	-1.147972	-11.10699	-0.030272
-9.019637	-0.476662	-32.10942	-1.030916	-10.33317	-0.117031
-9.400117	-0.359942	-31.41492	-0.796596	-10.40895	-0.055292
-8.760797	-0.508497	-32.07826	-0.796596	-9.974963	-0.082552
-8.461318	-0.59763	-31.04245	-1.080381	-10.05074	-0.020693
-8.501958	-0.54027	-30.85695	-0.991487	-10.12652	0.041227
-7.862917	-0.601692	-31.01129	-0.845768	-9.692538	0.01415
-8.073477	-0.514501	-30.14613	-0.870238	-9.08893	0.049237
-7.774277	-0.51638	-29.96064	-0.78113	-8.825163	0.143935
-7.475356	-0.430827	-30.45407	-0.809505	-8.731023	0.176533
-7.515997	-0.373106	-29.41973	-0.631076	-8.636882	0.209191
-7.217078	-0.287191	-28.89367	-0.829688	-7.863062	0.123177
-6.578038	-0.348121	-29.048	-0.683616	-7.769217	0.248165
-6.1092	-0.29155	-28.01293	-0.736077	-7.334942	0.129434
-5.6398	-0.410379	-28.33644	-0.79282	-7.411018	0.284346
-5.340602	-0.411708	-28.6607	-0.618236	-6.807414	0.320771
-4.871483	-0.442683	-27.2858	-0.727283	-6.543652	0.416801
-5.082327	-0.266799	-27.1003	-0.637666	-6.449513	0.450067
-4.443571	-0.239205	-27.08472	-0.51961	-5.506071	0.42774
-4.484496	-0.092548	-26.04965	-0.571687	-5.071796	0.309022
-4.015379	-0.123095	-26.03333	-0.685154	-4.638115	0.375981
-3.716183	-0.123876	-26.18768	-0.538586	-4.544275	0.50254
-3.077429	-0.095552	-25.66235	-0.505411	-4.280218	0.506553
-3.288277	0.08153	-24.79645	-0.760696	-3.846241	0.48095
-2.47932	-0.008055	-25.12145	-0.353749	-4.261865	0.603795
-2.350329	0.109983	-23.74655	-0.462165	-3.657968	0.548638
-2.221054	0.139391	-23.90015	-0.547252	-3.05437	0.586891
-2.431904	0.317191	-24.05375	-0.632339	-2.960234	0.620948
-1.283109	0.168576	-23.69908	-0.338422	-2.356338	0.566038
-1.154405	0.376508	-23.17302	-0.536946	-2.432423	0.72348
-1.025131	0.406339	-22.98753	-0.4466	-2.168667	0.821813
-0.556589	0.555593	-22.46295	-0.180623	-1.565073	0.861039
-0.427317	0.585666	-21.93688	-0.379127	-0.451418	0.717541
-0.12841	0.675692	-22.09122	-0.231852	-0.697425	0.9055
0	0.975251	-21.05615	-0.283002	-0.603291	0.940224
1.488057	0.948138	-20.86992	-0.424781	0	1.07443
1.277199	1.128333	-20.68443	-0.334071	0.604195	0.926074
1.915074	1.431396	-19.98919	-0.328353	1.037262	1.184989

2.044343	1.462496	-19.46387	-0.294206	1.640851	1.225798
2.683373	1.40458	-19.9573	-0.322559	1.565669	1.10152
2.812932	1.345195	-19.60264	-0.027228	1.489881	1.16646
2.941621	1.557811	-19.41566	-0.401745	2.262783	1.367485
3.070017	1.861929	-19.061	-0.106266	2.697058	1.248813
4.048594	1.83621	-18.53568	-0.071824	2.621572	1.219145
3.328261	2.016421	-17.84045	-0.065656	3.055239	1.290356
4.305667	2.35672	-18.5038	-0.065656	3.318986	1.391476
4.9444	2.39147	-17.80856	-0.059412	3.752347	1.55844
5.072784	2.699182	-16.60358	-0.138133	4.186316	1.535109
5.711515	2.734659	-16.75792	0.009996	4.620591	1.416448
5.50094	2.826699	-16.23186	-0.188426	4.884335	1.518296
5.969454	2.984443	-15.8772	0.108045	5.147466	1.811704
6.097831	3.294551	-15.86088	-0.005331	6.431342	1.544737
6.395828	3.668689	-16.01523	0.143009	6.355856	1.515074
7.03366	3.984964	-14.8095	-0.168767	6.280064	1.581163
7.843198	3.713837	-14.96459	0.213158	6.544419	1.492175
7.632619	3.806893	-14.60844	0.04311	6.80816	1.594874
8.101123	3.967707	-13.57413	0.226926	7.241206	1.860317
9.079686	3.946618	-13.72698	-0.091741	8.184625	1.845098
8.018906	4.375422	-13.71216	0.262263	7.93922	1.845098
9.336707	4.483076	-13.18609	0.063893	7.863426	1.911731
9.975426	4.522432	-12.15103	0.014297	8.636925	1.92654
9.594322	4.834142	-12.13546	0.134802	8.73105	1.963695
10.40266	4.938682	-12.2898	0.283713	9.504856	1.882278
10.53161	5.068021	-11.42466	0.26268	9.259142	1.978872
10.49065	5.22727	-11.57825	0.177675	9.863027	1.927237
11.63942	5.084459	-11.7326	0.326727	9.957153	1.964637
10.91878	5.362749	-10.35846	0.45504	10.73065	1.980306
12.06695	5.409768	-9.832393	0.256715	10.82415	2.211857
12.02598	5.56998	-10.32657	0.462643	11.59764	2.228135
12.32515	5.576007	-9.29076	0.17933	11.52216	2.19848
12.45349	5.897389	-8.935361	0.243679	11.61659	2.139171
13.09128	6.226299	-9.08896	0.158687	11.88033	2.244658
13.56007	6.298957	-9.243308	0.3081	12.65382	2.261427
13.68902	6.431183	-8.378162	0.287608	12.74763	2.397053
13.98757	6.629972	-7.513768	0.5018	13.01167	2.405651
14.62628	6.673816	-8.007198	0.473473	13.61399	2.843981
14.92545	6.680998	-6.802221	0.396557	14.38873	2.470308
15.39393	6.851215	-6.785904	0.283254	13.80411	2.334076
15.01249	7.266851	-6.940253	0.433022	14.40737	2.479213
15.8208	7.379012	-6.245024	0.441238	14.84101	2.556506
16.11935	7.580689	-5.549796	0.44953	14.76459	2.821146
16.58813	7.656129	-6.383809	0.712798	15.70831	2.711822

16.88761	7.567162	-5.688581	0.721234	15.80243	2.750803
17.01654	7.702157	-4.652769	0.438022	16.40568	2.897756
17.31478	8.002523	-4.637204	0.559825	16.83932	2.976264
17.95316	8.146882	-4.281808	0.625058	17.2736	2.857679
17.9128	8.117229	-3.926412	0.690364	17.19843	2.729453
18.38063	8.487526	-3.400348	0.492135	17.2916	3.064835
18.50956	8.624205	-3.555453	0.877827	18.23532	2.956139
19.6577	8.681428	-3.029388	0.67961	18.16015	2.827674
19.10789	8.642526	-1.654498	0.575245	17.91411	3.025373
19.74532	9.08438	-2.488514	0.839055	18.85719	3.114863
20.55393	9.103676	-1.623372	0.819786	18.95131	3.154696
20.51325	9.172709	-1.267223	0.649903	19.55455	3.304201
20.13179	9.597665	-1.420822	0.564962	19.64899	3.244917
21.62039	9.400196	-1.406013	0.923044	20.59238	3.236207
21.24019	9.429841	0.477868	0.969976	20.0065	3.494509
21.36816	9.866342	-0.014807	0.705828	21.11982	3.456646
22.00621	10.11584	0	1.064328	21.38386	3.467448
22.81545	9.937989	0.69598	0.837854	21.30773	3.63743
22.60453	10.1372	1.052128	0.667999	22.25081	3.729843
23.24321	10.18846	0.727859	0.847359	22.5158	3.440945
23.37181	10.42881	1.591488	1.300918	22.2704	3.440945
23.83993	10.7106	3.137046	0.932945	22.70339	3.722604
23.96884	10.85185	2.4737	0.932945	23.6471	3.615349
24.26736	11.06419	1.810354	0.932945	23.74218	3.355726
25.0766	10.88638	3.015325	0.857841	23.32525	3.886681
24.86567	11.08751	2.86097	1.009248	24.43856	3.850545
24.99394	11.43122	3.556194	1.019208	24.53267	3.891839
26.31229	11.36637	3.742426	0.877695	25.13687	3.743667
25.93209	11.396	3.927902	0.972641	25.2313	3.684398
26.2306	11.61033	4.453209	1.011057	25.15453	4.057624
26.69903	11.79556	4.637928	1.342805	26.26816	3.921426
26.99786	11.90948	5.673739	1.059814	26.02275	3.921426
27.29636	12.12525	5.010393	1.059814	25.94694	3.99272
27.76511	12.21015	5.365026	1.363548	27.06025	3.957697
27.55417	12.41379	7.079742	1.204257	27.32364	4.172305
28.5317	12.71657	6.756985	0.910793	27.58734	4.286047
28.32077	12.92105	6.262797	1.119368	27.34161	4.387453
29.12869	13.15299	7.127934	1.101644	28.28532	4.281516
29.25727	13.40163	8.332144	1.264485	28.38008	4.120789
30.23511	13.60564	6.819978	1.168881	28.64379	4.2349
30.19475	13.57602	8.533175	1.484197	29.07708	4.421332
30.83374	13.53091	8.550251	1.13382	29.85055	4.447167
30.79272	13.70745	8.565808	1.257864	29.94465	4.489802
30.92095	14.06125	8.921957	1.088115	30.3786	4.473247

31.7292	14.19395	9.786333	1.308133	29.79336	4.532499
31.85809	14.34196	9.292903	1.279843	30.90666	4.499573
32.49608	14.60907	10.66626	1.652829	31.00044	4.644645
32.79556	14.52023	10.51419	1.092926	31.77423	4.569149
33.09504	14.43138	10.19067	1.036348	32.20784	4.655337
33.39352	14.65457	10.88437	1.522721	32.64146	4.741769
33.69267	14.66986	11.91942	1.477554	32.90549	4.755454
33.6513	14.953	12.27557	1.307844	32.9996	4.798822
34.1197	15.14784	12.12045	1.698578	33.43321	4.885741
34.41884	15.16355	12.64651	1.500595	33.01723	5.121006
34.54773	15.31361	12.66283	1.387463	34.47036	5.03069
35.18671	15.27029	12.1694	1.359179	34.0557	4.854421
35.14501	15.6602	14.56225	1.564012	34.31972	4.868475
35.95358	15.69267	14.23873	1.507451	34.58342	4.985626
35.9122	15.97869	14.08437	1.660715	35.35687	5.014162
36.38093	16.07098	15.28858	1.826056	35.79115	4.895687
36.68007	16.08762	14.45608	1.616095	35.20458	5.368146
36.63869	16.37472	14.13256	1.559538	36.48746	5.412141
37.78709	16.34933	16.18634	1.583652	36.75214	5.219715
38.08657	16.26052	15.86283	1.527099	36.50674	5.219715
37.53578	16.5314	15.87838	1.652375	36.94002	5.412163
38.17408	16.70136	16.5736	1.664621	37.20404	5.427074
38.13168	17.3093	16.75983	1.523248	37.8079	5.382818
39.27973	17.39209	16.26716	1.256516	38.07159	5.501793
39.57921	17.3033	17.30068	1.689344	38.50587	5.383337
39.87835	17.32116	18.16657	1.434888	38.93914	5.577063
40.00722	17.47544	18.18213	1.560461	39.54367	5.324878
40.47594	17.57077	17.34887	1.588733	39.46785	5.399387
40.94465	17.66635	19.23273	1.642193	39.56128	5.652886
40.90326	17.95814	18.57014	1.403393	39.82497	5.772834
41.37128	18.26911	18.58493	1.767979	40.42849	5.833803
41.84068	18.15074	20.46879	1.821887	41.03202	5.895017
41.79963	18.33619	20.31443	1.976154	41.46562	5.986085
42.26765	18.64861	19.82176	1.708818	42.06982	5.838042
43.24681	18.44148	21.19587	1.847833	41.82441	5.838042
42.86661	18.47107	21.3821	1.706506	42.25869	5.719608
43.50489	18.64685	20.88791	1.91745	42.52237	5.840534
44.14421	18.49891	22.43193	2.02864	42.95597	5.932094
43.59305	18.88236	22.61817	1.887327	43.21999	5.94836
44.74109	18.97047	21.61576	1.704505	43.31341	6.204764
45.21014	18.96051	22.9891	2.083604	43.91761	6.056736
44.65967	19.12814	23.51593	1.646291	44.35188	5.938314
45.63813	19.13812	23.53071	2.012291	44.10614	6.043642
45.76595	19.62271	24.22592	2.025836	44.36948	6.27117

45.7249	19.81104	24.24224	1.912801	45.8226	6.18633
46.87397	19.57438	24.08787	2.067717	45.57719	6.18633
46.83291	19.76296	24.61394	1.869912	45.67129	6.232813
47.13169	19.89262	24.97008	1.700365	46.10387	6.64375
47.60004	20.10236	25.49461	1.982166	46.7084	6.389755
47.55898	20.29178	25.34024	2.137296	46.80284	6.330552
47.85705	20.64177	26.71435	2.27824	47.06651	6.453792
48.66629	20.4643	25.54279	1.883001	47.83995	6.488474
48.62593	20.43472	26.40792	1.868683	47.42462	6.518074
48.75478	20.59529	27.10313	1.882697	48.19873	6.340475
48.71266	21.11607	27.62842	1.925039	48.29249	6.493804
50.54142	20.76118	27.47329	2.320702	48.89634	6.452164
50.16122	20.79075	29.01808	2.193763	48.99044	6.499382
50.1198	21.09212	28.18558	1.981699	49.42369	6.700597
51.09754	21.3272	28.03198	1.896948	49.6877	6.718461
51.39666	21.34916	29.23617	2.06691	50.12129	6.813567
51.35559	21.54112	29.59155	2.137876	50.21538	6.861211
51.9942	21.61503	29.77701	2.237161	50.81855	7.034104
51.95348	21.69644	30.64136	2.46419	51.42274	6.886132
51.91277	21.7779	29.97879	2.223535	51.68573	7.22602
52.72094	21.93396	30.33417	2.294722	52.46018	6.941203
52.84978	22.09743	31.70904	2.196519	52.21512	6.834003
54.16806	22.05418	31.89527	2.055301	52.30887	6.989277
53.44767	22.2544	31.23115	2.296101	52.91203	7.163455
54.59567	22.35278	32.60525	2.438995	53.34596	7.152589
54.55567	22.21152	32.62234	2.085094	53.77955	7.249407
54.51459	22.40541	32.29805	2.26955	54.04356	7.268318
55.15319	22.48129	33.16317	2.256393	54.30791	7.179547
55.28132	22.87027	33.85838	2.271553	54.23174	7.365502
55.92028	22.83464	33.87315	2.640949	54.83558	7.325419
56.21832	23.19527	34.39922	2.44329	55.26917	7.422972
56.34824	23.02373	34.92528	2.245631	55.70309	7.412664
56.81763	22.90549	34.43185	2.217394	56.30659	7.480997
56.94575	23.29641	34.78722	2.28926	55.89058	7.727093
57.24379	23.65873	35.82148	2.48968	56.66434	7.657922
58.39286	23.42227	35.32883	2.220024	56.92801	7.786036
58.18258	23.42227	35.3436	2.589997	57.53151	7.855225
57.8013	23.7907	36.71846	2.492759	57.62629	7.687469
58.77864	24.14982	36.22504	2.464526	58.06056	7.56913
59.24768	24.14498	36.41049	2.564993	58.1543	7.727199
59.71708	24.02677	38.29433	2.624993	58.24769	7.994338
60.01619	24.05155	37.80168	2.354997	59.53121	7.836984
60.14502	24.21951	37.98636	2.69745	58.94597	7.896149
60.78361	24.29906	38.68156	2.713525	59.54947	7.9662

61.25264	24.2946	38.86857	2.330481	59.6425	8.34368
61.38147	24.46317	38.03453	2.600615	60.24635	8.305091
61.85051	24.45889	40.2582	2.604778	60.51105	8.107039
61.98006	24.39979	39.59486	2.604778	61.11489	8.068453
61.59914	24.65737	40.11937	2.891489	61.20898	8.118669
61.89715	25.0255	40.81612	2.423516	61.81212	8.299258
62.87521	25.16189	40.66175	2.581029	61.56671	8.299258
63.34461	25.0437	40.16832	2.552805	62.34012	8.341082
63.98356	25.0105	41.54163	2.941088	62.60413	8.362087
64.28267	25.03646	42.23761	2.715321	63.20762	8.433857
64.58141	25.17718	42.42229	3.059196	63.13178	8.514198
64.3704	25.40678	42.43938	2.703796	63.73597	8.36632
64.66877	25.66299	43.13458	2.720569	63.99892	8.717868
64.96751	25.80456	42.47123	2.720569	64.43249	8.820069
65.43581	26.03214	44.01524	2.838996	65.20695	8.532348
66.07475	25.99981	44.71121	2.61326	64.62135	8.701776
66.03402	26.0857	43.70804	2.669694	65.73425	8.796395
66.16321	26.14211	44.06263	2.985935	65.99754	9.039423
66.97096	26.42744	45.09844	2.703784	66.26189	8.950711
67.60991	26.39554	44.60579	2.432757	66.52695	8.640771
67.39926	26.5114	44.9596	2.992101	65.94171	8.699914
67.86791	26.62514	46.16532	2.681756	66.71476	8.854418
67.9971	26.6821	45.67112	2.896558	67.31824	8.928157
68.46576	26.7962	46.19562	3.185302	67.75181	9.03171
69.10396	26.99735	47.06229	2.688255	68.01652	8.832056
69.23426	26.70568	46.56886	2.660044	68.11024	8.994872
69.19315	26.90875	46.92267	3.220444	68.88364	9.039658
69.8321	26.87748	48.46823	2.853735	69.14729	9.173392
69.96017	27.2846	48.31463	2.76911	69.24172	9.114258
70.42919	27.28317	47.48137	2.797318	70.01548	9.048168
70.72829	27.31134	49.36519	2.861004	69.59979	9.189104
71.36723	27.2805	48.70185	2.861004	69.69352	9.352892
71.15658	27.39738	49.90601	3.037812	70.46692	9.398663
71.79515	27.48367	50.43129	3.084019	70.90047	9.503808
72.09388	27.62927	50.10778	3.027612	71.16376	9.750672
71.54263	28.04003	49.95418	2.943001	71.5966	10.08067
72.6902	28.27362	50.81851	3.176659	72.5417	9.537459
73.32877	28.36125	50.83561	2.820074	72.63578	9.59037
73.79779	28.36088	51.69994	3.053888	72.55994	9.672903
73.92622	28.65535	51.88539	3.156801	72.99385	9.666814
74.73545	28.47822	52.24076	3.231587	73.2582	9.578132
74.35563	28.38984	52.42699	3.090593	73.69212	9.572106
74.99457	28.36013	52.61244	3.193655	73.6152	9.992015
74.95307	28.68468	52.62954	2.836806	74.04874	10.09887

75.93185	28.59618	53.32317	3.343601	74.99277	9.891968
75.38173	28.65522	54.69802	3.249515	74.91729	9.86241
75.85037	28.77361	53.86477	3.277709	75.35156	9.744178
76.14871	29.04021	53.71116	3.193125	75.27572	9.827261
76.61735	29.1592	55.25594	3.070932	75.02923	10.16554
77.42545	29.33816	54.7625	3.042738	76.8225	9.92375
77.21516	29.33816	55.28622	3.578794	76.06734	10.01242
77.51389	29.48729	56.15211	3.325074	76.84036	10.1741
78.32236	29.54811	55.99851	3.240501	77.44383	10.25263
78.45039	29.96564	55.84491	3.155929	77.36835	10.22308
79.08894	30.05671	56.87994	3.118637	77.2925	10.30677
78.70874	30.08622	57.06538	3.222372	78.23544	10.43999
80.02736	29.94003	57.41996	3.542805	78.66936	10.43527
80.32684	29.85149	58.11594	3.317308	78.93371	10.34662
80.11579	30.0905	57.96234	3.232747	78.85786	10.43061
80.41412	30.36092	57.63804	3.421261	79.46169	10.39647
81.05228	30.57287	58.84298	3.356175	79.55576	10.45103
81.52168	30.45484	58.68938	3.271619	80.32915	10.50125
81.65047	30.63579	59.21466	3.319356	80.42358	10.44215
82.11871	30.87817	60.07977	3.310801	80.85676	10.66569
82.58811	30.76015	60.43434	3.632104	81.63014	10.71653
82.71729	30.82139	59.771	3.632104	81.21445	10.86023
83.01561	31.094	61.82473	3.671885	82.32803	10.73807
83.82484	30.91698	61.502	3.370153	82.25255	10.70852
83.95402	30.97847	61.85657	3.691891	82.00678	10.82279
83.57265	31.36982	61.87289	3.579177	82.95008	10.8447
84.38073	31.55521	62.22903	3.410107	83.38435	10.72652
84.6806	31.34584	62.07543	3.325572	83.13821	10.9555
84.81054	31.16604	63.10967	3.534899	84.2518	10.83372
85.27955	31.16883	62.95607	3.45037	83.83537	11.20736
85.06734	31.77374	63.31143	3.526968	84.77867	11.23026
86.04533	31.93091	64.17575	3.764906	85.38287	11.08255
86.00497	31.90141	64.53189	3.595865	85.13746	11.08255
86.47281	32.26915	64.54742	3.729032	85.23079	11.36847
87.11173	32.24326	64.56374	3.616342	86.00453	11.30634
87.41121	32.15477	65.59875	3.580553	86.77754	11.47484
87.54077	32.09578	65.7842	3.685698	86.8716	11.53112
88.01017	31.97779	65.46069	3.629356	87.4758	11.38343
88.30887	32.13266	66.83475	3.783528	87.57023	11.32435
88.43842	32.07367	66.68194	3.452879	87.83459	11.23573
88.22697	32.43916	67.20721	3.501841	87.92754	11.63873
89.37449	32.69136	66.71378	3.473673	88.36182	11.52058
89.33373	32.78406	67.57809	3.712659	88.62617	11.43196
89.97305	32.6366	67.93424	3.543659	89.06007	11.42949

89.93191	32.85167	67.44002	3.761844	89.15414	11.48615
90.22981	33.25304	68.13521	3.782939	89.58766	11.59964
91.20858	33.16924	69.17023	3.747784	89.51218	11.57011
90.9983	33.16924	69.18654	3.635129	90.45547	11.59525
91.46691	33.29669	69.71102	3.931175	90.37962	11.68175
91.76521	33.57679	70.23708	3.734042	91.15335	11.62064
92.40413	33.55233	70.2534	3.621395	90.90795	11.62064
92.70322	33.58691	69.25022	3.677719	91.68132	11.67581
92.83277	33.52794	70.7942	3.805097	91.60471	11.99529
93.30099	33.77948	70.98122	3.417522	92.54837	11.90503
94.11022	33.60257	71.16587	3.770342	92.47326	11.75904
93.55931	33.90815	71.52043	4.095273	92.90642	11.99047
93.68729	34.34314	73.06519	3.97628	92.83056	12.07758
94.83517	34.47839	71.38394	3.651195	93.43437	12.04663
94.62529	34.35463	72.41737	4.11077	94.03782	12.13257
94.92437	34.38995	73.45318	3.82922	93.79204	12.24945
95.5625	34.6142	73.4687	3.963788	94.73608	12.04275
96.20142	34.59081	73.3151	3.879327	94.31963	12.42329
95.99113	34.59081	75.0282	4.227157	95.77267	12.36233
96.28982	34.75056	74.70547	3.923462	95.69718	12.33281
97.09747	35.07079	74.38196	3.867158	95.79161	12.27375
97.22583	35.3853	74.73731	3.945647	95.88492	12.56662
97.69482	35.39201	75.94224	3.883458	96.65828	12.62439
98.33374	35.3693	75.44881	3.855308	97.09218	12.62385
98.4637	35.18569	75.97329	4.153404	97.35616	12.65288
98.76278	35.22194	76.83918	3.900072	97.78967	12.77019
99.06226	35.13352	76.8547	4.035158	98.56416	12.47532
99.19101	35.32409	76.19135	4.035158	97.97929	12.4167
99.66001	35.33106	78.07515	4.108524	98.41282	12.53401
100.1294	35.21317	77.58251	3.832561	99.0155	12.85794
99.91913	35.21317	78.27611	4.351055	99.44978	12.73986
100.3877	35.34517	78.80296	3.906096	99.54383	12.79885
100.8563	35.47742	78.47945	3.849808	99.63789	12.85791
100.8152	35.69832	78.66489	3.95705	100.4112	12.91717
101.2833	35.95645	79.86981	3.895511	100.6752	12.94689
101.7519	36.08955	80.05524	4.002903	101.1091	12.94716
102.2217	35.84617	80.2391	4.606891	101.0332	13.03604
102.5208	35.88329	81.10499	4.353646	101.6367	13.12543
102.3097	36.1345	80.44323	3.857105	101.5604	13.33314
103.1178	36.33499	79.9498	3.828964	102.5041	13.24523
103.2469	36.40194	81.15234	4.512799	102.5985	13.1862
104.0553	36.4771	82.18815	4.231436	103.032	13.30573
104.3548	36.38871	82.37358	4.339268	103.4663	13.18767
104.1433	36.76707	81.71103	4.090755	103.7295	13.45596

104.6111	37.15456	83.2542	4.470748	104.1638	13.33791
105.4191	37.35743	83.10139	4.137695	104.2578	13.39795
105.8885	37.2396	83.62666	4.189415	104.6917	13.39903
105.6774	37.49298	83.30154	4.630658	104.6151	13.72727
106.3163	37.47248	85.18613	4.457549	105.7287	13.61055
106.6154	37.51096	84.6935	4.180556	105.6532	13.58103
107.2547	37.36367	84.36998	4.124293	106.0875	13.46299
107.5534	37.52919	84.72453	4.453309	106.3514	13.49389
107.5126	37.62676	85.92946	4.392891	106.2748	13.82304
107.8117	37.66548	85.43682	4.115757	107.0481	13.8854
108.2807	37.67482	85.79058	4.694203	107.4824	13.76737
108.7501	37.557	86.9963	4.384812	107.4069	13.73786
108.3695	37.71367	87.35165	4.465268	108.1803	13.80047
109.0076	37.9484	86.85822	4.437143	108.1044	13.89088
109.4766	37.95804	87.89323	4.405176	109.048	13.80431
109.435	38.31132	88.41849	4.457661	108.8015	14.16461
109.9035	38.44896	88.60392	4.566467	109.5756	13.98758
111.0522	38.34119	87.77065	4.594589	109.6693	14.16908
110.8411	38.597	88.97638	4.285242	110.2727	14.2623
111.8195	38.64692	88.31223	4.534737	110.1976	14.1124
111.2689	38.83394	89.85619	4.668358	110.4612	14.26475
111.9078	38.81488	90.72128	4.664984	111.2349	14.20826
112.2065	38.98309	90.56767	4.580629	111.3286	14.39049
112.1649	39.3389	89.90433	4.580629	111.7625	14.39319
112.9741	39.16224	91.27837	4.742826	112.0264	14.42546
113.1029	39.36049	90.95645	4.186971	111.7802	14.66718
113.7426	39.08467	90.63133	4.63036	112.8934	14.67314
113.7018	39.18383	91.66634	4.599111	112.9878	14.61414
114.1704	39.32346	92.53143	4.596058	112.9124	14.58464
114.4686	39.62167	91.69817	4.624173	113.5162	14.55822
114.9376	39.63287	93.41203	4.730754	113.7805	14.46972
115.2367	39.67358	93.76898	4.311951	114.0429	14.9875
115.5357	39.71435	93.10403	4.81227	115.3264	14.84347
116.1746	39.6963	93.79921	4.837639	115.0806	14.96497
116.4741	39.60799	94.6651	4.584644	115.5145	14.96854
116.6028	39.8076	93.49281	4.418709	115.7785	15.00167
116.9015	39.97801	94.18638	4.944826	115.7018	15.3374
117.1993	40.40788	95.73273	4.329005	116.8154	15.22331
117.6691	40.1605	95.40842	4.523198	116.9106	14.92066
118.308	40.14296	95.59304	4.883677	117.0042	15.10534
118.0982	40.01332	96.96707	5.047692	117.0983	15.16827
117.8862	40.53223	96.30533	4.546399	117.5306	15.66082
119.2044	40.52715	96.48995	4.907169	118.4746	15.45439
119.164	40.49771	97.01601	4.710437	118.2296	15.33216

119.633	40.50998	97.71038	4.987181	118.8338	15.18471
119.9308	40.9423	97.38686	4.930976	118.9275	15.37025
120.0604	40.88343	98.42107	5.151802	119.191	15.52654
120.5285	41.1568	98.77802	4.732236	119.7948	15.50156
121.1674	41.14014	98.62441	4.647932	119.8893	15.44258
121.4669	41.05186	98.30009	4.842696	119.9833	15.50614
121.0867	41.08128	99.67413	5.007634	120.4172	15.51078
121.7252	41.1953	100.0303	4.839043	120.6812	15.54497
121.8543	41.26711	100.2149	5.200964	120.7748	15.73148
122.4924	41.51234	100.571	5.032384	121.5477	15.92325
122.9609	41.65652	101.2662	5.058926	122.1507	16.14506
122.9193	42.02037	100.7744	4.528264	122.585	16.02712
123.7273	42.23764	100.959	4.890352	122.1697	16.05661
123.8568	42.17879	101.8233	5.140335	123.2825	16.19052
124.3262	42.06111	102.3493	4.94368	123.3773	16.00823
124.4549	42.26511	101.686	4.94368	123.4705	16.31941
124.7532	42.57157	102.7202	5.165869	124.5845	16.08356
125.2217	42.71735	102.5674	4.829999	124.3387	16.20706
125.0114	42.71735	103.091	5.388346	124.0925	16.45425
126.1601	42.61381	105.1455	5.190717	125.0357	16.49532
125.6091	42.93647	104.6529	4.910823	125.8102	16.19469
126.9273	42.93577	103.4798	4.995092	125.3945	16.34792
125.867	43.21497	104.5148	4.966009	125.9971	16.69612
127.5249	43.15582	105.2092	5.245127	126.6009	16.67278
127.6545	43.09699	106.584	5.160107	126.6957	16.48978
127.9536	43.14105	105.241	5.272449	127.1296	16.49592
128.0823	43.34701	106.4451	5.467744	127.3932	16.65578
128.5508	43.49438	106.463	4.851166	127.3173	16.75053
128.6795	43.70083	106.4777	5.243076	128.2609	16.6685
129.1489	43.58318	106.8331	5.326799	128.0151	16.79285
129.4484	43.49495	107.6981	5.326352	127.9392	16.88779
130.0869	43.61341	107.2047	5.298271	128.543	16.8649
130.8957	43.5698	108.4096	5.241744	129.146	17.09132
130.1752	43.79094	108.0853	5.438016	129.5803	16.97343
130.3022	44.53085	108.952	4.932859	129.8439	17.13452
131.9606	44.34071	108.7976	5.101062	130.2777	17.14147
132.0906	44.14856	109.6611	5.606082	130.3714	17.33239
131.8799	44.28189	110.8668	5.29724	130.2951	17.55302
132.0086	44.48993	110.0343	5.072667	131.5789	17.2878
132.1377	44.56464	109.5401	5.29724	131.6726	17.47921
133.2868	44.3294	111.2539	5.409685	131.7666	17.54554
132.9053	44.75972	110.932	4.848018	132.3696	17.77436
133.5438	44.88028	111.7955	5.353728	132.9738	17.62703
134.0119	45.1645	111.1313	5.606652	133.0682	17.56809

134.3114	45.07629	112.5053	5.77584	133.5025	17.45023
134.9507	44.92929	112.8631	5.101348	133.2563	17.70131
134.57	45.09275	112.538	5.551276	133.8593	17.93106
135.5488	45.02108	113.064	5.354783	134.8029	17.85062
135.8482	44.93288	114.2681	5.552365	134.5575	17.85062
135.9769	45.14251	113.2658	5.355294	134.9914	17.85864
136.1056	45.35239	113.791	5.412023	135.2549	18.02218
136.5742	45.50372	114.4862	5.44076	135.5189	18.05985
136.7029	45.71409	115.1813	5.469575	135.9523	18.1943
137.8515	45.61359	115.0269	5.638795	136.5561	18.17324
137.131	45.83651	115.8912	5.89325	136.1404	18.329
138.2793	45.871	116.2482	5.471311	137.2543	18.09331
138.2393	45.70675	116.4336	5.584552	137.348	18.28718
138.1981	45.94712	115.7702	5.584552	137.2717	18.51075
139.3472	45.71197	116.9751	5.529491	138.2145	18.68453
139.1369	45.71197	116.6516	5.473367	138.4784	18.72294
139.4355	45.89381	117.685	5.95406	138.7424	18.76142
139.3943	46.13468	118.5509	5.701525	139.3466	18.61414
140.0323	46.39357	117.8883	5.447682	138.9312	18.6436
140.5013	46.41141	118.2429	5.787081	139.1952	18.68214
140.9711	46.15844	119.4494	5.224518	139.9681	18.8865
141.2693	46.47667	118.4462	5.280636	140.0617	19.08195
141.9081	46.46531	119.1397	5.818131	140.6655	19.06195
141.697	46.73666	120.3455	5.509512	141.2701	18.78741
142.3359	46.72549	119.3423	5.565625	141.0247	18.78741
142.1252	46.86132	120.0375	5.595306	141.6276	19.02216
143.103	47.06347	120.9025	5.59701	142.0611	19.15933
143.0631	46.89808	120.24	5.342809	142.3246	19.32619
143.7015	47.02324	121.2734	5.825078	142.5882	19.4933
143.8306	47.1006	122.3092	5.544556	142.8517	19.66065
143.7885	47.61635	122.6645	5.630656	143.4563	19.38552
144.9367	47.65422	122.5101	5.800982	143.5503	19.45451
144.8964	47.62484	123.0361	5.604631	143.9838	19.59266
145.0255	47.70264	122.5427	5.57658	144.2469	19.88869
145.3245	47.75112	124.0858	5.9758	145.0206	19.84026
145.4536	47.82904	124.272	5.835564	145.2858	19.49549
146.0921	47.95568	123.9477	6.034225	145.3798	19.56479
146.9009	47.91627	123.964	5.92204	145.3035	19.7919
146.8596	48.1608	125.1689	5.868365	145.9064	20.02996
147.329	48.04329	124.8454	5.812275	146.8497	20.08102
147.798	48.06283	125.2015	5.644005	146.6047	19.95241
147.4165	48.5037	125.726	5.957569	147.2089	19.80519
148.3943	48.71012	126.4219	5.733225	147.3029	19.87492
148.8641	48.45521	125.5879	6.016308	147.0566	20.13233

149.3331	48.47513	126.9627	5.934917	147.6596	20.37169
149.2919	48.72076	126.8091	5.850794	147.5837	20.47122
150.1002	48.81977	127.6733	6.108863	148.5273	20.39418
150.2302	48.62337	128.7083	6.083796	148.6209	20.59356
149.8491	48.92811	128.5555	5.744357	149.3942	20.67543
150.4876	49.05687	127.7215	6.02772	149.4886	20.61655
150.7861	49.24461	129.0963	5.946663	149.9217	20.88701
151.0861	49.01854	129.2825	5.806478	150.1848	21.18745
151.5541	49.31512	129.2972	6.205331	150.4487	21.22885
151.6815	49.94768	129.9923	6.236653	150.7127	21.27031
152.4898	50.04843	130.5184	6.040416	150.8058	21.60109
152.1096	50.07779	130.0258	5.756773	151.7495	21.52507
152.7481	50.20815	130.5502	6.071824	151.674	21.49564
153.0466	50.39749	130.9063	5.903627	151.9375	21.66754
153.3443	50.86497	130.7519	6.075278	152.5404	21.91114
154.1536	50.6888	132.2958	6.222575	152.8044	21.95322
154.1123	50.9376	132.4821	6.082426	153.0683	21.99536
154.4109	51.12791	131.8187	6.082426	153.3318	22.16812
155.0498	51.12041	132.6838	6.086121	153.9356	22.15159
155.3488	51.17168	132.87	5.945978	154.1995	22.19399
155.6469	51.50202	133.5643	6.233886	153.4431	22.67465
156.1159	51.52419	133.4099	6.405971	154.5558	22.83217
156.5848	51.54642	134.6165	5.841512	155.1591	22.9473
157.0542	51.42899	133.2718	6.466025	155.5938	22.69845
156.6736	51.59806	135.4962	6.245919	155.5179	22.80019
157.1425	51.62042	134.8337	5.989606	155.4412	23.1646
157.6106	51.9227	135.8678	6.222068	156.0441	23.41187
158.0795	51.94531	136.5622	6.510861	156.6475	23.52799
158.3781	52.13747	136.7484	6.370758	156.5707	23.89386
158.8475	52.02007	135.916	6.142256	157.5135	24.08376
158.6368	52.16027	136.7794	6.659842	158.4571	24.00991
158.5955	52.4115	137.8152	6.379656	158.3824	23.71642
159.7441	52.31709	138.0014	6.239563	158.1366	23.84843
160.2135	52.19969	137.5072	6.468278	159.0798	23.90671
160.0024	52.48064	138.2023	6.500941	159.5128	24.1858
159.9611	52.73249	138.5593	6.076033	160.1166	24.1711
160.4287	53.17821	138.5739	6.477652	160.0419	23.877
161.4069	53.25471	139.9488	6.398416	160.1359	23.95049
161.7064	53.16668	139.6261	6.085436	160.7393	24.06823
162.1763	52.90831	139.131	6.571388	161.0032	24.11249
162.3049	53.13167	141.0147	6.665433	160.9265	24.48092
162.6035	53.32593	140.692	6.352254	162.2095	24.48176
162.9025	53.37914	140.5384	6.268217	161.9641	24.48176
162.8617	53.49109	140.723	6.642546	161.8882	24.58521

163.3302	53.6565	142.097	6.821085	163.1716	24.45345
163.6288	53.8515	141.7743	6.507704	163.2664	24.26171
164.0977	53.87574	141.9597	6.625027	162.8499	24.69007
164.5667	53.90005	142.8248	6.630394	163.7935	24.61731
165.0351	54.0662	143.0118	6.232912	163.8875	24.69167
165.5045	53.94885	142.3468	6.747866	163.981	24.89936
165.4628	54.34523	144.4005	6.815001	164.7547	24.85628
166.4424	53.99791	143.9079	6.529348	165.1894	24.6053
165.8923	54.05658	143.9225	6.932696	165.1139	24.5759
166.1899	54.5368	144.9592	6.394939	165.0376	24.81325
167.1677	54.75828	144.2958	6.394939	166.3198	25.08287
167.2964	54.98446	145.1592	6.916293	166.4146	24.8904
167.7658	54.86713	146.0259	6.406416	165.998	25.32095
167.7254	54.8378	145.5325	6.378414	167.2797	25.72612
168.5333	55.08953	146.9057	6.816219	168.0547	25.28162
168.492	55.34565	146.7529	6.474214	167.4699	25.20646
168.6202	55.71563	146.0895	6.474214	167.9037	25.22282
169.5998	55.36739	146.6139	6.794307	168.3376	25.23924
169.8988	55.42235	148.1586	6.688487	168.7719	25.12164
170.3687	55.16209	147.8351	6.632493	168.0154	25.61227
170.4973	55.38939	148.1896	6.98102	169.4675	25.8557
170.9672	55.12907	148.5466	6.55476	169.9022	25.60367
170.5856	55.58759	148.5629	6.442776	169.8262	25.70872
171.0541	55.75672	148.4085	6.617087	170.6004	25.53233
171.692	56.0402	149.2735	6.623503	170.6948	25.47354
171.9915	55.95223	149.9687	6.657993	170.7879	25.81846
172.1201	56.18062	149.4753	6.63	171.3908	26.07575
172.7594	56.03401	150.5094	6.867161	171.8242	26.22798
173.0584	56.08965	150.6956	6.727208	171.9187	26.1692
172.6773	56.40638	151.0509	6.817912	172.3525	26.18662
173.3148	56.83531	152.2558	6.768748	173.4665	25.95148
173.9536	56.83275	152.2721	6.656794	172.8816	25.87526
174.0827	56.91821	152.1177	6.831619	172.9748	26.22166
174.892	56.74231	152.3031	6.950539	173.9184	26.1511
175.0211	56.82783	152.6593	6.78262	174.1832	25.92774
175.1506	56.7692	153.0146	6.873634	173.9373	26.06293
175.2793	56.99907	153.5398	6.936737	174.8805	26.12775
175.9176	57.14123	153.726	6.796815	174.6342	26.39854
176.3861	57.31296	154.2513	6.86	175.238	26.38709
176.1749	57.60219	154.0968	7.035191	176.0112	26.48192
177.3235	57.5124	155.3025	6.727385	175.9349	26.72393
177.2832	57.48309	154.6384	6.986599	176.0293	26.66516
177.9211	57.77102	155.8432	6.938093	176.8025	26.76049
178.2206	57.68308	156.3685	7.001588	177.2372	26.50709

178.1793	57.94373	156.7238	7.093138	176.8215	26.67233
178.4778	58.14601	156.2303	7.065159	177.2549	26.82668
178.9467	58.17397	157.2662	6.785378	178.368	26.86375
179.2453	58.37662	156.7727	6.757399	178.6319	26.91176
180.0536	58.49159	157.467	7.052652	178.3856	27.18426
179.8428	58.63709	158.3321	7.060508	178.9893	27.17369
180.3118	58.66543	158.0077	7.264263	179.5931	27.16319
180.6108	58.72313	158.3639	7.096419	179.3481	27.02678
181.2501	58.57661	159.2289	7.104435	179.9506	27.42586
181.3797	58.518	158.9054	7.048489	180.5543	27.41561
181.1684	58.80943	159.0908	7.168477	180.4789	27.38623
181.6369	58.98389	160.125	7.408669	180.7423	27.57158
182.4452	59.09999	160.8218	6.924915	181.8554	27.61028
182.2345	59.24604	159.6478	7.268819	181.9494	27.68848
183.0441	58.92418	161.3625	7.137309	181.8739	27.6591
182.6626	59.39182	161.7186	6.969496	182.3077	27.67861
183.9806	59.42064	161.2235	7.461865	182.911	27.80594
183.7699	59.567	162.2593	7.182197	183.0059	27.61005
183.7276	60.12371	162.4447	7.30264	183.2694	27.79627
185.216	59.97741	162.2911	7.218743	183.5329	27.98273
185.1752	60.09483	162.6472	7.050949	184.307	27.80648
185.9845	59.91906	163.0025	7.14351	183.7217	27.86523
186.1145	59.71375	163.6969	7.440705	185.0047	27.8757
186.0736	59.83118	163.7123	7.589409	184.9292	27.84633
186.2027	59.91937	164.7481	7.309793	184.683	28.1214
186.8411	60.06665	163.9157	7.077192	185.796	28.16175
186.6304	60.21362	164.7799	7.346735	186.4002	28.01489
187.4391	60.18489	165.306	7.151012	185.4747	28.27013
187.058	60.50842	165.4914	7.271918	186.4174	28.47815
188.0362	60.59786	166.0158	7.59782	187.1906	28.57799
187.9953	60.7159	166.7117	7.374161	187.1147	28.68674
187.954	60.98145	166.0492	7.113313	187.718	28.81632
189.2735	60.5704	166.7435	7.411421	188.1531	28.42243
189.0637	60.423	167.2696	7.215727	187.2276	28.67808
188.8539	60.27567	166.6062	7.215727	188.1716	28.47251
189.1534	60.18779	166.4526	7.131858	188.2661	28.41378
189.4538	59.80542	166.8079	7.225118	187.341	28.53124
189.4134	59.77613	165.8064	6.759102	187.9452	28.38441
188.8633	59.83471	166.1609	7.113297	188.3803	27.99063
189.673	59.5118	166.3471	6.973522	187.7947	28.18749
188.7845	59.18784	166.5325	7.094819	187.8891	28.12876
188.9135	59.27624	166.5472	7.505344	187.8145	27.8232
188.8732	59.24695	167.244	7.020518	187.5695	27.68519
189.0023	59.3354	166.4107	7.048471	187.1538	27.85257

189.1328	58.98279	166.427	6.936659	188.0969	27.92319
189.2628	58.77728	166.2734	6.8528	187.682	27.81442
189.053	58.63041	166.6287	6.946304	188.1172	27.42087
188.8432	58.4836	166.4743	7.123735	188.3815	27.33277
189.483	58.19038	167.3402	6.872177	187.6264	27.42087
189.1032	58.07298	166.5069	6.900128	187.3801	27.69695
188.8929	58.07298	166.1834	6.844226	188.3245	27.35331
189.022	58.16108	166.3696	6.704472	187.3999	27.33277
188.9826	57.83846	166.5558	6.564719	187.6638	27.38267
188.9418	57.9558	165.7209	7.115468	188.268	27.23583
189.2412	57.86791	166.7559	7.097483	187.8536	26.98923
188.861	57.89721	166.6031	6.752134	187.948	26.93049
189.161	57.66268	165.9389	7.01364	188.042	27.00971
188.6109	57.72128	166.2951	6.845954	187.6267	27.03908
189.2511	57.2817	166.3114	6.734164	187.7207	27.11836
188.8705	57.45751	166.4959	7.117654	188.1549	27.00089
189.3403	57.19381	166.8512	7.211695	188.0794	26.97152
188.9601	57.2231	167.0383	6.810253	187.6637	27.13897
189.2605	56.84235	166.5449	6.782308	188.4378	26.96276
189.0507	56.69601	165.7116	6.810253	187.8539	26.60743
188.6705	56.72531	166.4076	6.58669	187.2686	26.66617
188.8005	56.52043	166.2523	7.026375	187.702	26.82468
188.4185	57.13521	167.4572	6.980863	187.9668	26.59855
189.3971	57.07665	166.7955	6.457195	187.5524	26.35205
189.3582	56.60796	166.3012	6.691054	187.6468	26.29331
188.9789	56.34463	166.3167	6.841152	187.7412	26.23457
188.9381	56.46162	166.6729	6.673498	187.8357	26.17583
189.4066	56.63717	167.1981	6.739848	187.0805	26.26394
188.5171	56.60796	166.3648	6.767789	187.6834	26.53099
189.3263	56.43217	166.7201	6.862155	187.9482	26.30486
189.117	56.13953	166.9072	6.460436	187.0231	26.42233
189.0771	55.964	165.5642	6.572201	187.9671	26.21675
188.6969	55.99331	166.2585	6.872778	188.062	26.02004
188.9949	56.34427	167.1244	6.621326	187.6471	25.9115
188.6147	56.37357	166.8008	6.565448	187.7411	25.99067
188.5734	56.63715	165.9668	6.855545	188.0054	25.90257
188.8729	56.54926	167.0025	6.576164	188.2702	25.67654
188.833	56.37349	166.679	6.520288	187.3443	26.06991
188.963	56.16848	166.1856	6.49235	188.1184	25.8937
189.2625	56.08059	166.0312	6.670772	187.7035	25.78509
188.5425	56.16848	167.0653	6.916081	187.4585	25.64717
189.3512	56.13911	166.5735	6.363466	187.8924	25.66761
188.9715	56.02199	165.3996	6.709582	187.9868	25.60888
188.5913	56.05129	166.6053	6.402288	187.5715	25.63824

188.2106	56.227	166.2818	6.346416	188.0049	25.79679
188.3388	56.60801	166.2964	6.759506	188.2705	25.29474
189.149	56.1391	166.8216	6.826487	187.176	25.30373
188.5984	56.34423	166.499	6.508102	187.6094	25.46203
188.7279	56.28563	166.3445	6.686824	187.7038	25.40329
189.3668	56.28575	166.8714	6.228766	187.6283	25.37392
189.157	56.13914	166.717	6.407497	187.0422	25.70868
188.9481	55.6997	166.3926	6.614228	188.3256	25.58248
188.567	56.0219	166.7488	6.446639	187.9116	25.19771
189.0359	56.05125	166.7651	6.334913	187.4958	25.36506
188.4857	56.10985	166.1009	6.597582	187.9301	25.24759
188.7838	56.46194	166.796	6.636877	187.8542	25.35627
189.2537	56.19804	167.153	6.206555	187.7782	25.46501
189.0434	56.19804	165.6401	6.346207	188.0417	25.6533
189.1734	55.99278	166.675	6.329653	188.4773	25.12132
188.7923	56.31547	166.3498	6.799487	187.8921	25.18006
189.6029	55.69969	167.0466	6.313182	187.6467	25.18006
188.713	55.81688	166.0435	6.369039	188.4203	25.14196
188.8425	55.75829	166.3988	6.464358	187.836	24.92453
188.8012	56.02226	166.4151	6.352649	187.4207	24.9539
188.5914	55.8756	166.6005	6.475974	188.0253	24.66906
188.89	56.0811	166.9566	6.308417	187.61	24.69843
189.5302	55.64123	166.803	6.224638	187.3637	24.97448
189.1509	55.37736	165.799	6.543521	187.6276	25.02449
188.6004	55.5825	166.664	6.555296	187.8919	24.93639
188.5591	55.84649	167.021	6.124644	187.8169	24.7689
188.8576	56.05212	166.1869	6.415676	187.4011	24.93639
188.9867	56.14038	166.5431	6.248131	188.1752	24.76018
189.4575	55.58282	166.8992	6.080587	187.4196	24.98646
188.9078	55.49474	166.235	6.343768	188.1928	25.0868
189.0379	55.28954	166.5903	6.43948	188.4581	24.72216
189.1674	55.23094	167.2863	6.216105	187.3631	24.869
187.9371	55.55334	166.6238	5.952848	187.796	25.16643
188.2357	55.75886	165.7888	6.507346	188.2303	25.04897
189.5551	55.34846	166.8246	6.228137	187.6459	24.83104
188.6657	55.31897	166.3312	6.200217	187.7395	25.04897
189.4744	55.28987	166.5166	6.32401	188.0052	24.54596
188.9252	55.05516	166.8727	6.156492	188.1	24.34904
188.0348	55.31897	166.3793	6.128572	187.3444	24.57533
189.3533	55.20198	166.5647	6.252446	187.9482	24.56674
188.2939	55.20178	166.581	6.140771	187.1921	24.93151
188.7628	55.23128	165.9185	5.877295	188.476	24.66722
188.3817	55.55411	166.1039	6.001178	188.0611	24.55821
188.3399	55.96558	166.1193	6.153051	187.6462	24.44927

188.8089	55.99545	166.6445	6.221245	187.7402	24.52885
189.1093	55.61354	166.8308	6.081663	187.8347	24.47012
187.3688	56.1712	165.9967	6.373263	187.7587	24.57912
189.0267	56.14325	167.2015	6.32995	187.3434	24.60849
188.9868	55.9668	167.0488	5.982447	187.7777	24.49102
188.6066	55.99609	166.0456	6.038277	187.5323	24.49102
188.7361	55.9375	166.7399	6.342549	187.7957	24.67985
189.375	55.93821	166.7562	6.230896	188.0601	24.59176
187.9739	56.5851	166.7725	6.119243	187.1346	24.84777
188.9525	56.52748	165.9393	6.147156	188.0777	24.91951
189.4229	56.11556	166.4645	6.215668	188.0036	24.4743
188.8728	56.17414	166.8206	6.048195	187.4183	24.53303
188.3222	56.38007	167.0068	5.908634	187.8521	24.55413
189.3023	55.8804	166.1727	6.200524	188.286	24.57529
188.4114	56.2922	166.6996	5.741161	187.5313	24.52477
188.2011	56.2922	166.0354	6.005146	187.6257	24.46604
189.0103	56.11646	165.881	6.185463	187.8909	24.10089
189.4802	55.85195	166.746	6.198394	187.1358	24.18898
189.1004	55.73395	166.5924	6.114667	188.0793	24.12193
189.0605	55.55742	166.4388	6.03094	187.3237	24.34858
188.8507	55.41025	165.7755	6.03094	186.9084	24.37795
189.1521	54.73426	165.7918	5.919304	188.0228	24.00447
187.9223	54.91003	165.4691	5.599353	186.4194	23.82406
187.3727	54.82175	164.8049	5.863487	186.5129	24.04216
188.6931	54.11782	164.9903	5.988143	187.118	23.6185
187.1248	53.91246	165.1774	5.584398	186.3646	23.15367
186.915	53.76601	164.0051	5.403984	184.9293	23.49738
187.5562	53.03429	163.3401	5.932328	186.0437	23.1243
187.007	52.80063	164.3759	5.65325	185.2903	22.66043
186.1189	52.33405	163.0329	5.764881	184.7059	22.44354
186.08	51.86753	163.0492	5.65325	184.2911	22.33518
186.2109	51.37225	162.3858	5.65325	184.3859	22.13878
184.3019	51.51988	161.7234	5.389037	183.8025	21.64746
184.9422	51.08265	161.5697	5.305311	182.5375	21.82371
185.0726	50.73357	161.5852	5.457896	182.9714	21.84364
184.5235	50.50197	161.2617	5.40208	183.2366	21.48072
183.8048	50.15503	161.4479	5.262542	181.2929	21.49992
184.1061	49.48809	159.7651	5.429988	182.0665	21.46092
182.8773	49.37484	160.2903	5.498925	181.9919	21.15711
182.6679	49.08595	159.6278	5.234635	181.5779	20.77529
183.1382	48.68002	159.814	5.095097	180.6528	20.8928
182.0797	48.39407	159.1498	5.359392	180.5765	21.13749
181.7005	48.13533	159.1662	5.247766	181.0125	20.47208
182.3416	47.41332	158.1638	5.039282	179.2383	20.59966

180.9428	47.33123	158.0102	4.955559	180.3531	20.09116
180.9038	46.87135	157.5159	5.191953	179.7683	20.01328
181.2047	46.35335	158.0412	5.260978	178.5042	19.91645
179.8059	46.27225	157.2087	5.024514	178.9385	19.79893
180.2757	46.01184	155.8657	5.13614	178.8643	19.36032
180.578	45.06649	156.5617	4.912888	178.4503	18.98103
179.1801	44.70144	156.4081	4.829169	177.3553	19.12795
178.9712	44.27429	155.235	4.912888	177.6192	19.17596
179.1016	43.93115	156.1001	4.926113	177.0357	18.69043
178.3843	43.16719	154.9278	4.74545	176.1106	18.80797
177.3253	43.03038	154.7742	4.661731	176.2047	18.88518
177.2859	42.71777	154.1109	4.661731	176.8097	18.46634
176.9061	42.60557	154.4662	4.758682	175.716	18.20586
177.207	42.09327	153.124	4.605919	175.1303	18.40038
176.3179	41.92809	153.6492	4.674966	175.565	18.14708
175.2585	41.93363	153.1558	4.647061	174.6404	18.12895
175.3885	41.73382	152.6623	4.619156	174.5662	17.69286
175.6889	41.36372	152.5087	4.53544	174.1509	17.72225
174.6299	41.22861	152.1852	4.47963	173.5665	17.5102
174.5905	40.91768	151.182	4.53544	173.6618	17.18082
175.2311	40.34901	151.8772	4.576665	173.077	17.1044
174.3425	40.04501	150.8748	4.368009	172.1528	16.95173
173.453	40.02206	151.4	4.437145	172.4167	16.99864
173.5835	39.68285	150.3969	4.492953	172.6823	16.50538
173.7139	39.34387	150.4132	4.381337	170.7382	16.66442
172.6563	38.79101	149.5799	4.409241	171.3424	16.51744
172.2765	38.68062	149.7653	4.534261	171.6076	16.15962
171.7268	38.59965	149.4426	4.213913	169.6634	16.31885
172.1962	38.48222	148.6094	4.241817	170.438	16.00781
171.6479	37.98279	148.285	4.450552	170.5332	15.67986
171.2686	37.73343	148.8119	3.990682	168.9285	15.91504
170.549	37.68225	146.6202	3.977339	169.3628	15.79745
170.1693	37.5724	147.6551	3.962778	169.4576	15.60417
170.64	37.0377	147.5007	4.14362	168.5334	15.45297
169.5814	36.76779	147.0072	4.115717	168.1181	15.48237
169.5415	36.59964	146.3439	4.115717	168.7231	15.06682
169.6724	36.1249	146.3594	4.268727	167.29	14.73623
168.6143	35.7179	146.2066	3.920397	167.3844	14.67743
168.2345	35.60897	144.8636	4.032008	167.1395	14.54348
167.8552	35.3619	145.3897	3.836689	166.7254	14.1714
167.4755	35.25314	145.0661	3.780884	165.2901	14.51099
167.2665	34.83912	143.5532	3.920397	166.0634	14.60228
166.8867	34.7306	144.0784	3.989709	166.1591	14.14199
166.507	34.62215	143.4159	3.725078	165.4044	14.0965

166.1263	34.78934	143.4314	3.878102	164.6492	14.18471
166.5975	34.12091	142.938	3.850201	165.2538	13.90404
165.8788	33.79644	142.7844	3.766496	164.3296	13.75454
165.1596	33.60981	142.46	3.975396	163.7439	13.94688
164.7794	33.63919	141.7975	3.710692	164.3494	13.39945
164.9094	33.44315	140.9651	3.473954	163.7655	13.0584
164.3606	33.0904	140.9797	3.891694	162.3315	12.99798
163.641	33.04148	142.0155	3.612688	162.5945	13.30925
163.6007	33.0121	140.8424	3.69639	162.8606	12.68848
162.8815	32.82633	140.0083	3.989066	161.5961	12.73194
163.1814	32.60132	140.3653	3.556887	161.3503	12.86493
162.4622	32.41591	139.5329	3.320071	161.6151	12.64371
162.5931	31.94726	138.6988	3.612688	161.0315	12.17118
161.704	31.79184	139.224	3.682171	160.2759	12.39216
162.0044	31.43097	138.5615	3.417385	160.5411	12.03849
161.6246	31.32409	138.2371	3.626372	159.9567	11.83212
161.0758	30.97441	137.9145	3.305784	158.8617	11.97919
160.5257	31.03318	137.2511	3.305784	158.957	11.65541
159.8061	30.98531	137.7763	3.375278	158.7116	11.65541
160.4458	30.70241	136.9422	3.668035	158.467	11.39069
160.2373	30.15913	136.9594	3.29158	158.0521	11.28784
159.5186	29.84047	136.1261	3.319479	158.1466	11.22901
159.3096	29.43418	135.8017	3.528544	157.2215	11.34667
158.4205	29.28119	136.1579	3.361154	156.9752	11.61126
158.5509	28.95209	135.1556	3.152083	157.5807	11.06738
157.322	28.85837	134.6613	3.389052	156.9971	10.59798
157.4524	28.52975	135.0166	3.4866	155.222	10.9947
157.2425	28.39492	134.863	3.402908	156.3356	10.89139
156.8632	28.15483	134.5412	2.817293	155.9215	10.5249
156.4843	27.78046	132.8583	2.984688	154.8261	10.8039
156.4444	27.61659	132.8738	3.137968	155.2608	10.55432
155.8951	27.40663	133.738	3.416845	155.0166	10.15895
155.3454	27.33114	133.0755	3.151834	153.5826	10.10162
155.3059	27.03334	131.9024	3.235525	153.847	10.01336
155.0965	26.76518	131.9195	2.858987	153.7715	9.983937
154.5472	26.55606	131.935	3.012349	152.8468	9.970073
153.6581	26.406	131.4416	2.984452	152.7722	9.677736
153.6182	26.24285	130.7782	2.984452	152.8675	9.356214
153.9181	26.02094	130.7945	2.872864	151.2631	9.460352
152.8591	25.90077	129.7922	2.663701	151.6966	9.605213
153.1594	25.54559	129.9767	3.054197	152.3016	9.195542
152.2699	25.52983	129.9931	2.942613	150.6981	9.037548
152.4003	25.20441	130.0102	2.565998	150.6218	9.270318
151.8514	24.86377	128.4973	2.705482	150.7162	9.21147

150.7916	25.01082	128.3428	2.886821	150.3021	8.84769
151.0906	25.05568	129.0388	2.663654	149.3771	8.965391
150.3706	25.14391	127.8666	2.482307	149.6418	8.746167
151.1815	24.43545	127.0316	3.040334	149.5676	8.324254
150.1229	24.18413	128.0666	3.026555	147.9624	8.690437
149.5732	24.11029	127.235	2.524175	148.9064	8.48445
149.5333	23.94827	126.4017	2.552071	148.8314	8.324254
148.9827	24.1397	126.0774	2.761386	147.7372	8.210035
149.2835	23.65381	126.0937	2.649807	147.8325	7.890061
148.5643	23.47725	125.6003	2.621912	146.9082	7.746901
148.1849	23.24211	124.9369	2.621912	146.4933	7.645984
147.8056	23.0072	125.1223	2.747618	146.4174	7.746901
147.7661	22.7137	124.9695	2.398755	146.0025	7.645984
146.8761	22.83137	123.7947	3.01286	145.2469	7.864621
146.6658	22.83137	124.4916	2.524469	145.8515	7.587123
146.6267	22.40628	122.9795	2.398755	144.418	7.402458
146.0775	22.20164	123.1649	2.524469	144.512	7.473762
145.8676	22.06999	123.5202	2.622363	144.7767	7.255299
145.3183	21.86571	123.7064	2.4829	143.3427	7.201171
144.5987	21.82251	122.3642	2.329214	143.6071	7.112873
144.8982	21.73424	121.7009	2.329214	143.7019	6.924019
144.179	21.55975	121.2075	2.30132	142.6073	6.941258
144.139	21.39904	121.5636	2.133959	142.7009	7.142305
143.7592	21.29723	120.7295	2.427115	143.306	6.735223
143.7202	20.87447	121.0848	2.525091	141.5321	6.740454
143.0009	20.70083	120.5922	2.231868	141.457	6.581273
142.6212	20.59938	119.4183	2.580874	141.7218	6.36328
142.4105	20.73026	119.9443	2.385634	140.7971	6.351395
142.2014	20.33782	119.4526	1.827132	140.5513	6.481022
142.3327	19.75656	118.6185	2.120298	140.9868	5.974573
141.6135	19.584	119.1428	2.4558	139.552	6.180631
140.8947	19.28148	118.4803	2.190395	139.8155	6.351395
140.6844	19.28148	118.6666	2.050938	140.0811	5.874561
140.1351	19.08011	116.474	2.30196	138.4768	5.980674
139.7549	19.10954	117.17	2.07883	138.9111	5.862923
139.8845	19.05067	117.1863	1.967265	138.4966	5.633766
139.5051	18.8201	116.6928	1.939373	137.5711	5.880787
138.6164	18.54829	116.1986	2.176898	138.0049	5.892361
138.4065	18.41856	116.2157	1.799917	137.5908	5.533998
138.3657	18.51885	115.3833	1.562453	136.6666	5.393523
138.1559	18.38912	115.0581	2.037447	136.9309	5.305202
137.9468	18.00029	115.0744	1.925886	136.5168	4.94774
136.8878	17.88856	115.2606	1.786435	135.4214	5.223853
136.8478	17.72975	114.2566	2.107706	136.1943	5.43429

136.468	17.62988	114.2729	1.996149	136.4599	4.958884
136.4277	17.60044	113.7803	1.702765	134.6852	5.223853
135.7081	17.5595	113.2869	1.674875	134.9496	5.13553
135.3275	17.71819	112.9625	1.884593	134.5355	4.778431
135.7968	17.60044	113.1488	1.745148	133.6112	4.63881
134.9073	17.58894	112.3155	1.773037	134.215	4.620261
134.358	17.38951	111.9928	1.451755	133.6302	4.55048
134.488	17.20155	111.3286	1.717259	133.0462	4.223726
134.109	16.84414	111.175	1.633592	132.6308	4.253171
133.8992	16.71531	111.0214	1.549925	132.0456	4.312061
133.5198	16.48727	110.6971	1.759722	131.7998	4.440546
133.1404	16.25948	110.3735	1.703946	132.0649	4.095299
133.1009	15.97303	109.7111	1.43837	131.1399	4.21308
132.3812	15.93296	109.3867	1.64817	130.7249	4.11416
132.1714	15.8046	109.5738	1.243148	131.1596	3.86807
131.792	15.57753	108.5689	1.830151	130.235	3.857609
130.902	15.69532	109.4348	1.57917	129.8192	4.0153
131.3722	15.32125	108.6015	1.607057	129.9145	3.699975
130.8233	14.99617	107.939	1.341405	129.33	3.502677
130.1037	14.95665	107.1049	1.634943	128.405	3.620469
129.7235	14.9861	107.631	1.439736	129.1791	3.443782
129.5132	14.9861	107.3074	1.383963	128.0837	3.719086
129.6436	14.67164	106.3034	1.705457	128.0078	3.817764
129.0947	14.34765	107.1684	1.720278	128.2733	3.345223
128.0352	14.3674	106.1678	0.978865	127.0088	3.393969
128.5046	14.24959	105.1638	1.300303	127.2724	3.561573
128.2952	13.99474	105.0093	1.482371	127.0282	3.177736
127.576	13.8285	105.5345	1.552971	126.2743	2.882788
127.0254	14.01468	105.042	1.259287	125.8589	2.912239
126.8156	13.88741	103.868	1.60874	125.9538	2.725691
126.6061	13.63305	104.225	1.17563	125.0287	2.843496
126.2263	13.53542	103.9015	1.119859	124.613	3.000591
125.8469	13.31088	103.7479	1.036203	125.2176	2.725691
125.296	13.62379	102.9138	1.329894	123.9526	2.902398
125.5967	13.15451	102.9301	1.218356	124.0471	2.843496
125.7274	12.71522	102.7765	1.134703	124.3122	2.500029
124.1582	12.82422	101.4343	0.980432	122.877	2.833717
124.1178	12.79476	101.4498	1.134703	123.4816	2.558934
124.0779	12.63869	101.2962	1.05105	122.8968	2.490373
123.5278	12.69761	101.4816	1.177512	122.481	2.64729
123.3187	12.31814	100.6483	1.205395	122.4063	2.362968
122.5991	12.28015	100.3248	1.149629	121.3113	2.510233
122.729	12.09492	100.1721	0.800091	121.7456	2.392421
122.5196	11.84248	98.99811	1.149629	121.6709	2.108336

121.63	11.8342	100.3729	1.080989	120.4064	2.157837
121.4193	11.96033	99.20065	0.898679	120.3314	2.001218
120.6997	11.92259	99.04704	0.815029	119.7465	1.933024
121.1699	11.55266	97.53327	1.2204	120.0109	1.844659
120.4502	11.5151	98.73898	0.913695	119.2561	1.805981
120.7505	11.17498	97.73581	0.96946	118.8404	1.96248
119.8614	11.04137	97.24238	0.941578	118.9348	1.903569
119.4808	11.19654	97.08878	0.857931	118.3508	1.581529
118.9315	11.00412	97.10424	1.012437	117.2554	1.855734
119.0614	10.81959	96.78072	0.956674	118.1998	1.522616
118.5113	10.87853	96.11823	0.690638	117.4446	1.610986
118.6413	10.69406	96.13368	0.84515	116.859	1.796821
117.7525	10.43571	95.64111	0.551228	117.1238	1.581529
117.2024	10.49465	94.63708	0.873031	115.8592	1.631407
117.3323	10.31042	94.8233	0.733626	116.463	1.610986
116.9525	10.21466	94.83877	0.888213	116.3884	1.327861
116.4032	10.02332	93.83559	0.943973	115.123	1.631407
116.023	10.05279	93.34216	0.916093	115.2171	1.699356
115.8123	10.1779	93.35847	0.804573	115.142	1.543036
115.7724	10.02332	93.20486	0.720933	114.0482	1.310098
115.393	9.802754	92.71059	0.959233	113.9731	1.154018
115.5237	9.369199	92.38707	0.903475	113.898	0.997996
114.4642	9.391816	92.57414	0.497894	113.1425	1.212936
114.0841	9.42129	91.9108	0.497894	113.5771	0.968537
114.3843	9.083548	91.75635	0.680445	111.9728	1.077712
113.6651	8.922894	91.60274	0.596808	112.4071	0.959873
112.7755	8.916348	91.10931	0.568929	112.5019	0.77451
113.2449	8.798444	90.2752	0.863064	111.0666	1.107172
112.6956	8.608677	90.97117	0.640042	111.8403	1.056915
112.4853	8.608677	90.47774	0.612164	111.4258	0.83343
111.2555	8.785538	89.47456	0.66792	110.1609	1.010192
111.7253	8.543362	89.8307	0.500654	110.5952	0.892351
111.176	8.35396	89.50718	0.444898	111.0299	0.648127
110.9657	8.35396	88.16418	0.556409	109.9353	0.669109
110.4164	8.164801	88.18049	0.444898	110.0297	0.610187
110.0358	8.318307	87.68706	0.417021	109.9546	0.454463
109.9962	8.040832	87.19363	0.389143	107.8401	0.778541
109.4465	7.975884	87.54892	0.488219	108.4443	0.631232
109.0671	7.757728	87.2254	0.432466	108.709	0.416646
108.8568	7.757728	86.73196	0.404589	107.4441	0.593418
108.4774	7.539813	85.89956	0.166122	107.3686	0.563956
108.6073	7.357241	85.40527	0.404589	107.8029	0.446108
108.0584	7.045739	85.76141	0.23733	106.7075	0.719617
106.9985	7.193156	84.58833	0.32096	107.3125	0.319966

106.6183	7.22264	84.94361	0.420117	106.7273	0.378891
107.0881	6.981338	84.79001	0.336491	105.6327	0.400125
106.3689	6.823234	84.63726	-0.013558	106.0669	0.282273
105.6484	7.034935	83.46332	0.336491	105.652	0.185716
105.6081	7.005451	83.47963	0.224989	105.2367	0.215179
106.0775	6.887513	83.49594	0.113487	104.9917	0.089219
105.0192	6.542308	82.66268	0.141363	103.8963	0.362496
104.8093	6.419301	81.82857	0.435726	103.4814	0.266002
104.2588	6.60128	82.18556	0.001986	104.2555	0.089219
104.3887	6.419301	82.03111	0.184857	102.6511	0.199033
104.0093	6.202953	81.53767	0.156983	103.0854	0.081176
103.6295	6.109613	81.21416	0.101234	102.6709	-0.140978
102.9099	6.07531	81.06055	0.017612	101.9153	0.073196
102.5296	6.104798	80.39635	0.284177	101.5004	-0.023117
102.6596	5.923115	79.73301	0.284177	101.9346	-0.140978
101.9403	5.766349	80.08915	0.116938	100.3299	0.094744
101.7293	6.011581	79.59572	0.089065	100.9341	-0.052582
101.8592	5.829957	78.76246	0.116938	100.5191	-0.148834
101.6493	5.70737	79.28851	-0.078174	100.1038	-0.119368
101.2703	5.369458	78.79423	0.160595	99.6885	-0.089903
100.3811	5.242789	78.47157	-0.161793	99.78294	-0.148834
100.0005	5.394567	77.63831	-0.13392	99.36801	-0.245026
100.1305	5.213297	77.99445	-0.301158	98.61285	-0.156627
99.58115	5.027888	76.82136	-0.217539	98.53777	-0.31169
98.86111	5.116366	76.49699	-0.006637	98.46228	-0.341157
98.65083	5.116366	76.17347	-0.062381	97.36768	-0.319359
98.61047	5.086874	76.3597	-0.201741	97.80195	-0.437227
98.40058	4.964768	75.1866	-0.118125	97.55654	-0.437227
98.19109	4.720735	75.54189	-0.018636	96.1217	-0.230957
97.81205	4.38463	75.72812	-0.157991	96.55638	-0.474302
97.26193	4.44362	74.89485	-0.13012	96.31097	-0.474302
96.71181	4.502611	74.06074	0.164538	95.38589	-0.356431
96.16169	4.561602	74.24867	-0.508332	95.14049	-0.356431
95.95102	4.683406	73.41456	-0.213733	95.0654	-0.511314
95.74113	4.561602	73.09019	-0.002682	94.30984	-0.297496
95.70155	4.288676	73.61624	-0.19777	93.72459	-0.238561
95.32134	4.318172	73.29272	-0.25351	94.32919	-0.511314
94.94154	4.226044	71.77981	-0.114161	93.57402	-0.42291
94.3918	4.163473	71.96603	-0.25351	93.49854	-0.452378
94.1823	3.920525	71.98235	-0.364989	93.42345	-0.607201
93.9724	3.79914	71.319	-0.364989	92.32804	-0.334506
93.42268	3.736812	71.16454	-0.181727	92.42288	-0.518795
92.87255	3.79581	71.52068	-0.348939	92.34739	-0.548264
92.66188	3.917133	70.00777	-0.209596	91.76255	-0.61462

92.45199	3.79581	69.68425	-0.265333	91.34722	-0.585151
91.90303	3.491198	69.87048	-0.404676	91.27174	-0.61462
91.86267	3.461698	68.52747	-0.293202	90.34666	-0.496744
91.14302	3.429116	68.71284	-0.165602	89.93133	-0.467276
90.93235	3.550198	69.57873	-0.41641	90.366	-0.710384
90.89238	3.399615	68.57555	-0.360675	89.61124	-0.747148
90.68249	3.278592	68.25204	-0.41641	88.85567	-0.533568
90.13275	3.216631	67.41877	-0.388542	89.28995	-0.651445
90.09239	3.187129	68.45457	-0.667217	89.04494	-0.776618
90.05241	3.036724	66.26114	-0.149395	87.94994	-0.629269
89.16245	3.154731	66.27745	-0.260861	88.04437	-0.688209
89.12286	2.883603	67.14333	-0.511659	87.62945	-0.78385
88.57391	2.58062	66.31007	-0.483793	87.0442	-0.724909
88.1937	2.610124	65.47681	-0.455926	86.96832	-0.629269
87.64281	2.910395	66.00287	-0.650991	87.23307	-0.84279
87.60245	2.880892	65.16961	-0.623125	85.62831	-0.607028
87.05349	2.578094	64.84609	-0.678857	85.72275	-0.665969
87.01351	2.428107	64.01198	-0.383895	85.9875	-0.87943
86.46339	2.487116	64.70795	-0.606818	85.06242	-0.761548
85.5742	2.364355	64.21452	-0.634683	84.98693	-0.791018
85.87445	2.035294	62.87151	-0.523222	84.5716	-0.761548
85.66455	1.915111	63.05774	-0.662549	84.32619	-0.761548
84.60468	2.062646	63.41388	-0.82974	84.08119	-0.886537
84.22409	2.212333	62.57891	-0.267465	83.66547	-0.732077
84.35365	2.15332	62.42701	-0.88547	83.25054	-0.827595
84.48282	2.214548	62.27256	-0.701886	82.49538	-0.739181
83.59363	2.092153	61.61007	-0.969066	82.25037	-0.864108
83.55404	1.822464	60.77595	-0.674022	82.17448	-0.768652
83.17461	1.61203	61.13209	-0.841207	81.249	-0.525782
82.45418	1.820496	60.12806	-0.518234	81.51416	-0.864108
82.41343	1.910988	59.80454	-0.57396	81.77851	-0.952523
82.20354	1.790988	59.48103	-0.629686	80.34327	-0.621296
82.33348	1.61203	59.66725	-0.769002	80.94746	-0.768652
81.44428	1.490365	60.02339	-0.93618	80.1927	-0.805165
81.06409	1.519874	58.51047	-0.796865	79.43753	-0.71675
81.19364	1.460855	58.35601	-0.613133	79.02221	-0.687279
80.30407	1.459134	58.71301	-1.047631	79.11664	-0.746222
80.09455	1.219795	57.37001	-0.93618	78.87124	-0.746222
79.88389	1.339435	57.38632	-1.047631	78.45591	-0.71675
78.82439	1.36735	57.57169	-0.919614	78.72026	-0.805165
78.78326	1.577175	57.41808	-1.0032	77.45574	-0.7532
78.74367	1.308328	55.56448	-0.540776	77.04041	-0.723728
78.53415	1.06923	56.26131	-1.031062	76.62469	-0.569392
77.98441	1.008796	55.76788	-1.058923	77.73904	-0.930032

77.77375	1.128254	55.61427	-1.142509	76.13388	-0.569392
77.56385	1.008796	54.78016	-0.847245	76.56855	-0.812144
77.01296	1.306795	55.1363	-1.01441	75.98331	-0.7532
76.80345	1.06782	54.64201	-0.774799	75.05823	-0.635311
76.59355	0.948422	54.14858	-0.802659	75.15306	-0.819059
75.87389	0.917623	53.99498	-0.886238	74.90725	-0.694256
76.00269	1.097332	53.67146	-0.941958	74.83176	-0.723728
76.13301	0.799571	53.17802	-0.969818	73.90668	-0.605839
75.58327	0.739319	53.02442	-1.053397	74.51128	-0.878004
75.03315	0.798346	52.87082	-1.136976	73.75611	-0.789587
74.14281	1.035675	52.71722	-1.220554	72.83103	-0.671696
74.61297	0.679128	51.54327	-0.869428	73.09538	-0.760114
74.57261	0.649615	51.89941	-1.03658	72.85037	-0.884857
73.85295	0.618999	51.91572	-1.148014	71.75497	-0.612751
73.47237	0.76767	50.91255	-1.092297	70.99981	-0.524334
73.09293	0.558932	49.90937	-1.03658	71.77432	-0.825911
73.05257	0.529417	49.92569	-1.148014	70.33948	-0.6196
72.50282	0.46941	49.43225	-1.175873	70.60383	-0.708019
72.46246	0.439895	49.10873	-1.23159	71.0381	-0.825911
71.23305	0.498012	49.29411	-1.103254	69.77318	-0.649073
71.53252	0.409465	49.1405	-1.186826	69.6973	-0.553806
71.1527	0.320065	47.96656	-0.835563	69.45269	-0.803226
70.43304	0.289759	48.32355	-1.270399	68.52721	-0.560654
70.22314	0.170964	47.66021	-1.270399	69.1314	-0.708019
70.01248	0.289759	47.67652	-1.381828	68.54615	-0.649073
69.63266	0.200481	46.67249	-1.058414	67.62107	-0.531181
69.25246	0.229998	46.85871	-1.197696	67.20574	-0.501708
68.70272	0.170298	45.85554	-1.141984	67.80994	-0.649073
68.83228	0.111264	45.70193	-1.225553	66.3755	-0.567439
68.28254	0.051625	45.54747	-1.041351	66.63985	-0.655859
67.73241	0.11066	45.56379	-1.152772	66.9042	-0.744279
68.03227	-0.096507	44.90044	-1.152772	65.97912	-0.626386
67.65208	-0.066989	44.91675	-1.264193	65.73371	-0.626386
66.76249	-0.067469	45.10212	-1.135622	65.99847	-0.839425
66.72213	-0.096988	44.60955	-1.431324	65.2429	-0.626386
66.17239	-0.156443	42.75594	-0.968497	63.97798	-0.449545
65.79181	-0.008432	43.79259	-1.514889	64.41225	-0.567439
66.26159	-0.245	43.97797	-1.386309	64.16685	-0.567439
65.03179	-0.067886	42.63496	-1.274893	63.41168	-0.479018
64.31174	0.020671	42.4805	-1.090538	62.65652	-0.390598
64.27139	-0.008848	42.83836	-1.79344	63.26111	-0.662582
64.06149	-0.127279	41.15467	-1.358455	61.99579	-0.361125
63.34144	-0.03872	41.51081	-1.525579	61.9199	-0.265924
63.98114	-0.304689	41.3572	-1.609141	62.52409	-0.413289

62.75134	-0.12757	40.86292	-1.369068	61.76933	-0.449545
62.54106	-0.12757	39.68983	-1.285509	61.18409	-0.390598
62.16124	-0.216359	39.70614	-1.396921	61.44844	-0.479018
61.78066	-0.06853	39.55253	-1.48048	61.37295	-0.508492
61.91059	-0.24588	39.56884	-1.591892	59.93811	-0.302178
61.19056	-0.157319	38.22498	-1.212486	60.20246	-0.390598
61.15057	-0.305088	39.09087	-1.463153	60.12738	-0.544686
60.77037	-0.275567	38.59743	-1.491005	59.03237	-0.397317
59.88041	-0.157484	37.76418	-1.463153	58.61705	-0.367843
59.33067	-0.216629	37.26989	-1.222938	58.71188	-0.551344
59.45985	-0.157484	37.62688	-1.658116	58.46608	-0.42679
59.58978	-0.334714	36.79277	-1.362192	57.71091	-0.338369
59.03929	-0.157484	36.46925	-1.417893	57.63543	-0.367843
58.48954	-0.216629	36.65461	-1.289005	57.39002	-0.367843
58.44918	-0.24615	36.67178	-1.66855	56.9747	-0.338369
58.23928	-0.364277	35.49784	-1.316855	56.72929	-0.338369
57.68878	-0.187108	35.68406	-1.456103	56.6538	-0.367843
57.47888	-0.305234	35.70037	-1.567502	56.4088	-0.492395
57.2686	-0.305234	35.20694	-1.595352	55.14388	-0.315549
56.71885	-0.364257	34.20291	-1.271438	55.57815	-0.433446
55.6586	-0.098583	34.55991	-1.70675	55.16282	-0.403972
56.12875	-0.45274	33.72665	-1.6789	55.25726	-0.462921
55.57825	-0.275691	33.23322	-1.70675	53.82241	-0.2566
55.53827	-0.423218	32.90884	-1.494227	53.91685	-0.315549
54.64793	-0.187125	33.09507	-1.63347	54.1816	-0.528463
53.92827	-0.216561	32.26181	-1.605622	52.91628	-0.227125
54.05782	-0.275606	31.59846	-1.605622	53.18103	-0.440039
53.50771	-0.216561	31.27408	-1.393028	52.76571	-0.410564
53.2978	-0.334503	31.63108	-1.828409	52.35038	-0.381089
53.59728	-0.423072	31.47662	-1.643655	51.59482	-0.168177
52.87724	-0.334503	30.98319	-1.671502	51.68965	-0.351614
53.17672	-0.423072	31.16941	-1.810739	50.76457	-0.233715
51.94692	-0.245935	29.99632	-1.727197	50.68908	-0.26319
51.22688	-0.157366	28.82324	-1.643655	50.78391	-0.446568
52.03611	-0.334503	29.34929	-1.838587	50.36819	-0.292664
51.65629	-0.422863	28.855	-1.598063	49.95287	-0.26319
51.10579	-0.245935	29.38105	-1.792987	49.70746	-0.26319
50.72597	-0.334293	27.86728	-1.38532	49.80229	-0.446568
50.51569	-0.334293	28.5641	-1.876526	48.02721	-0.056866
49.45618	-0.304495	27.90076	-1.876526	48.6314	-0.20424
49.58574	-0.363543	26.89672	-1.552392	48.72623	-0.387617
49.37546	-0.363543	26.57321	-1.608083	48.14059	-0.20424
48.82497	-0.186676	27.09926	-1.802999	47.21591	-0.210765
48.44514	-0.274972	25.7554	-1.423109	47.48026	-0.299191

48.40478	-0.304495	25.77257	-1.802999	47.23486	-0.299191
47.68512	-0.333683	26.12785	-1.701551	45.96954	0.002084
47.47446	-0.215924	25.46536	-1.97007	47.42332	-0.292664
47.26455	-0.333683	24.80116	-1.701551	46.49824	-0.174765
46.88435	-0.304158	24.98738	-1.840771	45.57355	-0.18129
46.33423	-0.24511	24.49395	-1.868615	45.49807	-0.210765
46.12395	-0.24511	23.83061	-1.868615	45.76282	-0.423558
45.57421	-0.303758	22.99649	-1.572184	44.32758	-0.092864
45.70414	-0.480445	23.69331	-2.063523	44.42202	-0.151815
45.32394	-0.45092	21.83971	-1.600027	44.51645	-0.210765
44.43361	-0.215185	22.87551	-1.878457	43.42184	-0.187752
44.903	-0.333283	22.55113	-1.665479	42.8366	-0.1288
44.35288	-0.274234	21.88864	-1.934143	43.44119	-0.400484
43.63322	-0.303295	21.73418	-1.749005	42.51571	-0.158276
43.42294	-0.303295	21.9204	-1.888214	42.10038	-0.1288
43.55249	-0.362345	20.40663	-1.480276	42.53466	-0.246703
43.00237	-0.303295	21.10259	-1.703003	41.77949	-0.158276
43.13193	-0.362345	20.77908	-1.758684	41.36456	-0.253103
42.41227	-0.391345	20.11573	-1.758684	41.79884	-0.371008
42.03207	-0.36182	19.79221	-1.814366	40.70383	-0.223627
41.65187	-0.332294	20.14921	-2.250157	40.28851	-0.19415
40.9322	-0.361231	17.95578	-1.730844	40.21302	-0.223627
40.72155	-0.243718	18.82166	-1.98141	40.47738	-0.312055
40.68156	-0.390757	19.00703	-1.851799	39.38237	-0.164674
39.96152	-0.302179	18.34368	-1.851799	38.96705	-0.135198
39.75161	-0.419632	17.34051	-1.79612	38.72164	-0.135198
39.37104	-0.272654	17.52759	-2.204136	38.30632	-0.105722
39.33105	-0.419632	16.01381	-1.79612	37.89059	0.048054
38.95085	-0.390106	16.36995	-1.963157	37.81551	-0.105722
38.23081	-0.301527	16.55532	-1.833468	37.5701	-0.105722
38.19083	-0.448445	15.21318	-1.990997	36.64502	0.012183
37.30087	-0.330338	15.39854	-1.861307	36.73945	-0.046769
36.92029	-0.183422	15.58477	-2.0005	36.66436	-0.200486
37.7299	-0.477972	14.75064	-1.703706	35.73888	0.041659
37.17978	-0.418918	15.44661	-1.926406	36.51299	-0.135198
36.45974	-0.330338	14.27353	-1.842894	35.58831	-0.141532
36.75922	-0.418918	13.95087	-2.16753	34.66322	-0.023626
35.86926	-0.300811	13.45658	-1.926406	34.92758	-0.112056
35.31952	-0.359087	13.47289	-2.037756	35.19193	-0.200486
35.27915	-0.388614	12.80868	-1.768726	33.75709	0.005851
34.72866	-0.212231	13.33473	-1.96358	33.85192	-0.17728
34.85859	-0.388614	12.16164	-1.880071	33.26628	0.005851
34.47839	-0.359087	11.83899	-2.20478	33.36071	-0.053102
33.24897	-0.299191	11.51461	-1.991417	32.26571	0.094281

33.03868	-0.299191	10.85126	-1.991417	32.53046	-0.118326
33.16824	-0.358246	11.37646	-1.917163	32.45457	-0.023626
33.12788	-0.387774	10.20337	-1.833657	31.18965	0.153235
31.89808	-0.210608	10.38959	-1.972833	31.28448	-0.029895
32.02802	-0.38687	10.06694	-2.297616	31.37931	-0.212966
31.64781	-0.357342	9.062901	-1.972833	30.62375	-0.000418
31.09807	-0.415432	9.249126	-2.112009	30.54827	-0.029895
31.05733	-0.327814	9.094662	-1.926334	30.6427	-0.088849
30.84705	-0.327814	8.431316	-1.926334	29.71802	-0.095056
30.63677	-0.327814	8.276851	-1.74059	28.62262	0.176445
30.59641	-0.357342	8.293162	-1.851922	29.05729	-0.065578
30.21658	-0.444961	7.120073	-1.768423	28.64196	-0.036101
29.49617	-0.23923	7.816902	-2.260343	28.39655	-0.036101
29.79602	-0.444961	6.812866	-1.935421	27.98083	0.117491
29.07598	-0.356375	6.658401	-1.749601	27.73582	-0.006623
29.03562	-0.385904	6.674712	-1.860929	26.64082	0.140765
27.97574	-0.238262	6.012228	-2.130252	27.07549	-0.101199
27.93538	-0.26779	5.687849	-1.916593	27.16953	-0.036101
27.55555	-0.355345	6.04485	-2.352916	26.07452	0.111287
27.34527	-0.355345	4.531071	-1.944425	26.5088	-0.006623
27.13499	-0.355345	4.717296	-2.083584	25.41379	0.140765
26.75479	-0.325817	3.883174	-1.786358	24.99847	0.170242
26.37459	-0.296288	4.91897	-2.064667	25.9429	-0.160156
25.99476	-0.383781	3.745882	-1.981175	24.50805	0.046191
25.61493	-0.471215	2.913484	-2.222743	23.92281	0.105147
25.23436	-0.324723	2.758156	-1.76737	24.527	-0.042243
25.19437	-0.471215	3.624038	-2.017838	23.77184	0.046191
24.64425	-0.412155	2.111121	-1.87869	22.67644	0.31763
23.75429	-0.294037	1.788466	-2.203821	23.45134	-0.10728
23.54438	-0.410937	1.634	-2.017838	23.03562	0.046191
23.67394	-0.469997	1.820225	-2.156987	22.11014	0.288153
22.1043	-0.233756	0.647137	-2.073498	22.37489	0.075669
22.91354	-0.410937	0.663447	-2.184816	22.12988	-0.048323
22.70326	-0.410937	0.678896	-2.026587	20.86496	0.128548
22.32306	-0.381407	-1.003935	-1.859615	20.78947	0.09907
21.60302	-0.292817	-0.81771	-1.998759	20.54407	0.09907
21.56303	-0.439186	-0.460708	-2.435283	20.29827	0.223059
21.01254	-0.263287	-1.465608	-1.840459	20.05286	0.223059
21.31239	-0.468717	-0.76964	-2.06308	19.97738	0.193581
20.76189	-0.292817	-1.772815	-2.007425	19.22221	0.282015
20.38207	-0.380125	-2.265384	-2.304873	19.8268	0.010634
19.83195	-0.321064	-2.760541	-1.793395	18.90172	0.128548
19.45174	-0.291534	-2.233625	-2.257872	18.48639	0.158027
19.41138	-0.321064	-2.558005	-2.043833	18.41091	0.128548

19.54132	-0.496904	-3.56118	-1.98818	17.14559	0.429405
18.82128	-0.408311	-4.054611	-2.016007	17.24042	0.246462
18.44107	-0.37878	-4.038301	-2.127312	17.16533	0.093056
17.72103	-0.290187	-4.701646	-2.127312	15.90001	0.393855
17.85097	-0.465966	-4.515421	-2.266444	16.5042	0.246462
17.47039	-0.319718	-5.009716	-2.024505	16.2588	0.246462
17.26048	-0.436435	-5.842977	-1.996679	15.16379	0.393855
16.37052	-0.31831	-5.99658	-2.080155	15.0887	0.24045
16.33016	-0.347841	-5.640441	-2.247107	15.18314	0.181493
15.78004	-0.288778	-7.494049	-1.7825	14.59789	0.24045
15.57051	-0.522028	-6.117562	-2.386233	14.86264	0.028146
14.85009	-0.316838	-6.780908	-2.386233	14.277	0.210972
14.98003	-0.492496	-8.124774	-2.005094	13.69176	0.269929
14.08969	-0.257774	-7.938549	-2.144215	13.78619	0.210972
13.87978	-0.374367	-7.581546	-2.581008	13.37087	0.24045
13.32966	-0.315303	-9.435155	-2.116391	12.6161	0.205023
13.62914	-0.4039	-8.739187	-2.338984	13.05037	0.087105
12.73918	-0.285771	-8.72374	-2.180364	12.46473	0.269929
12.52927	-0.402303	-9.047258	-2.23601	11.70996	0.234502
12.48854	-0.315303	-9.881382	-1.938206	11.63487	0.081218
11.93842	-0.256238	-9.694293	-2.347302	10.87971	0.169657
11.55822	-0.226706	-10.18859	-2.105138	10.12455	0.258097
11.68852	-0.518775	-11.1909	-2.319479	10.89866	0.081218
11.1384	-0.459709	-11.34623	-1.862909	9.463418	0.411379
10.41836	-0.37111	-9.969742	-2.466823	9.727771	0.32294
10.20771	-0.25464	-12.84284	-1.835088	9.312843	0.228617
10.50756	-0.459709	-11.12652	-2.494644	8.387759	0.346536
9.957064	-0.284172	-12.30047	-2.141118	8.311877	0.440858
9.407688	-0.457987	-12.62313	-2.466823	9.086385	0.140177
8.177517	-0.164378	-13.62716	-2.141118	7.651147	0.470337
9.157418	-0.603872	-12.59137	-2.419326	7.406137	0.346536
8.437005	-0.39892	-13.76532	-2.065733	7.840806	0.104874
7.716965	-0.31032	-14.59772	-2.308043	6.745803	0.252275
8.016443	-0.39892	-14.24244	-2.204832	6.839841	0.317057
7.296775	-0.426669	-14.05535	-2.614071	6.934274	0.258097
6.576363	-0.221719	-15.73905	-2.177012	6.009191	0.376016
7.21568	-0.369387	-15.04395	-2.129364	6.443463	0.258097
6.835106	-0.223445	-15.36746	-2.185001	5.688299	0.346536
6.285358	-0.280786	-16.36977	-2.39957	4.763612	0.340716
6.075077	-0.280786	-16.69415	-2.185001	5.197884	0.222795
6.035088	-0.426669	-16.33715	-2.622127	4.442325	0.434976
5.31542	-0.454355	-17.85093	-2.21282	4.876597	0.317057
4.935219	-0.42482	-16.98505	-2.463187	4.631588	0.193315
4.385098	-0.365752	-17.64926	-2.192906	3.366665	0.370197

4.174817	-0.365752	-17.63209	-2.574461	2.950944	0.523416
3.964536	-0.365752	-18.63613	-2.248541	3.216089	0.187556
3.074948	-0.363839	-18.61895	-2.630098	2.460529	0.399677
3.204506	-0.422909	-18.77342	-2.443264	2.045204	0.429157
2.824677	-0.50954	-19.60668	-2.415446	2.309953	0.217036
1.933974	-0.159011	-19.76115	-2.228544	0.875111	0.423402
2.573663	-0.422909	-19.57406	-2.637985	1.819538	0.093416
1.683703	-0.30477	-21.42767	-2.172911	0.55422	0.393921
1.473793	-0.420934	-20.90075	-2.637985	0.308815	0.393921
1.263512	-0.420934	-21.05608	-2.180649	0.743087	0.275998
0.713392	-0.361864	-20.69908	-2.617972	-0.691755	0.482363
0.50311	-0.361864	-22.21286	-2.208464	-0.596926	0.299785
0.292829	-0.361864	-21.85586	-2.645788	-0.672016	0.146746
-0.087	-0.448433	-22.35102	-2.132674	-1.427576	0.358747
-0.636749	-0.505405	-22.67281	-2.729237	-1.672981	0.358747
-1.017321	-0.359826	-22.99805	-2.243931	-1.408628	0.270303
-0.887392	-0.534941	-22.81183	-2.383001	-3.013785	0.629767
-1.607804	-0.33029	-24.49553	-1.945553	-2.239278	0.329266
-1.818085	-0.33029	-24.13766	-2.653507	-2.654207	0.235191
-2.028366	-0.33029	-24.12221	-2.494258	-3.74921	0.382599
-2.238276	-0.446333	-24.95547	-2.466444	-3.145018	0.235191
-2.788396	-0.38726	-24.93916	-2.5777	-3.900182	0.323636
-2.998678	-0.38726	-24.7538	-2.446189	-4.145588	0.323636
-3.548798	-0.328188	-26.26672	-2.307124	-4.051549	0.388228
-4.098919	-0.269116	-25.91058	-2.474002	-5.146157	0.412081
-3.969361	-0.328188	-26.40401	-2.501815	-4.711489	0.170658
-4.51911	-0.385097	-26.89744	-2.529628	-5.636573	0.288586
-4.899311	-0.35556	-27.39174	-2.286793	-6.222212	0.471044
-5.109592	-0.35556	-26.86569	-2.481476	-5.957464	0.259104
-4.980034	-0.414633	-28.20956	-2.099515	-6.882943	0.500526
-6.039913	-0.266951	-27.51272	-2.592724	-6.958033	0.347551
-5.909984	-0.441943	-28.5159	-2.5371	-6.8636	0.288586
-6.630024	-0.353333	-28.32967	-2.676159	-8.128522	0.465479
-6.670386	-0.38287	-29.84345	-2.26638	-7.69425	0.347551
-7.560346	-0.264722	-29.14749	-2.488867	-8.10918	0.253599
-7.260868	-0.353333	-29.64179	-2.245885	-9.034658	0.494961
-7.471149	-0.353333	-30.13435	-2.544488	-8.430071	0.224117
-8.700948	-0.176111	-30.28795	-2.62792	-9.865308	0.553926
-8.4011	-0.38058	-30.10173	-2.766974	-9.43064	0.312565
-8.781301	-0.351042	-31.78543	-2.329314	-9.676046	0.312565
-9.331421	-0.291968	-30.91955	-2.579601	-10.43161	0.524443
-9.201493	-0.46684	-31.58376	-2.308734	-9.656704	0.100743
-9.921903	-0.262431	-31.90641	-2.635221	-11.43138	0.36609
-9.962265	-0.291968	-32.57062	-2.364351	-11.33695	0.307124

-10.68231	-0.203356	-32.21362	-2.802078	-11.24252	0.248158
-11.06213	-0.289614	-33.72827	-2.121227	-11.99808	0.459978
-10.93295	-0.232893	-32.86152	-2.642437	-11.73372	0.37153
